# Mining metastasis related genes by primary-secondary tumor comparisons from large-scale databases

**DOI:** 10.1186/1471-2105-10-S3-S2

**Published:** 2009-03-19

**Authors:** Sangwoo Kim, Doheon Lee

**Affiliations:** 1 Department of Bio and Brain Engineering, KAIST, 373-1 Guseong-Dong, Yu-seong Gu, Daejeon, 305-701, Republic of Korea

## Abstract

**Background:**

Metastasis is the most dangerous step in cancer progression and causes more than 90% of cancer death. Although many researchers have been working on biological features and characteristics of metastasis, most of its genetic level processes remain uncertain. Some studies succeeded in elucidating metastasis related genes and pathways, followed by predicting prognosis of cancer patients, but there still is a question whether the result genes or pathways contain enough information and noise features have been controlled appropriately.

**Methods:**

We set four tumor type classes composed of various tumor characteristics such as tissue origin, cellular environment, and metastatic ability. We conducted a set of comparisons among the four tumor classes followed by searching for genes that are consistently up or down regulated through the whole comparisons.

**Results:**

We identified four sets of genes that are consistently differently expressed in the comparisons, each of which denotes one of four cellular characteristics respectively – liver tissue, colon tissue, liver viability and metastasis characteristics. We found that our candidate genes for tissue specificity are consistent with the TiGER database. And we also found that the metastasis candidate genes from our method were more consistent with the known biological background and independent from other noise features.

**Conclusion:**

We suggested a new method for identifying metastasis related genes from a large-scale database. The proposed method attempts to minimize the influences from other factors except metastatic ability including tissue originality and tissue viability by confining the result of metastasis unrelated test combinations.

## Background

Cancer metastasis is spread of a tumor from its primary organ to other part or non-adjacent organs. During the last several decades, development of cancer treatment and surgical technology has greatly increased survival rate of cancer patients [1], but treatment of metastasis still remains above of medical capability. Once cancer cells have been disseminated to distant organs through lymph/blood vessels, they always have a potential for re-colonization to form secondary tumors. Furthermore, the newly generated tumors already have genuine ability to form second metastasis [2]. From these reasons, metastasis is the cause of about 90% of deaths from solid tumors. The biology of metastasis has been studied for more than 100 years since Stephen Paget first proposed the 'seed and soil' hypothesis [3]. During or after the complex genetic changes in normal cells' tumorigenesis, a small portion of tumor cells acquire additional abilities. It is generally accepted that a tumor cell has to go through a lot of obstructions and overcome harsh conditions [4,5]. For example, the new environment hardly supplies the metastasized tumor cells with hormones or ligands which are indispensable for cellular growth and proliferation. It means that metastasize tumor cells need to rearrange their genetic contents to live without those signalling proteins. Tumor cells also face with physical barriers including basement membranes (BM), extracellular matrices (EM) and vessel walls. In this case, some cells who secured higher motility, ability for detachment survival and ability to change their physical/biological characteristics through epithelial-mesenchymal transition (EMT) get favorable opportunities to win a competition among the other tumor cells and move on to the next metastasis barriers. There are many other chemical/physical barriers in whole metastasis procedures including intravasation (getting into a vessel), high fluid pressure in vessels, scattered immune cells, and extravasation (getting out from a vessel). Micrometastasis is a microscopic secondary tumor resulted from a set of primary tumor cell's success in hurdling all of the barriers above. Forming an outgrowing tumor in the secondary site is extremely hard because the entire hurdling events are a series of long odds. Even though a micrometastasis settles down in the new site, it usually dies from the inharmonious environment surrounding the cell or lies dormant due to the lack of suitable growth factors. So, the metastasized tumor cells in the secondary site have been chosen by selective pressures to have all the abilities for metastasis [6,7]. Sometimes, these winner cells are called 'decathlon champions'.

Many researchers have tried to explain metastatic procedures in the genetic level either in small scale experiments or from large scale expression data. Wang *et al *identified 76 gene signatures using 286 lymph node negative breast cancer expression data [8]. They used unsupervised clustering to classify good and bad prognosis. Tomlins *et al *tried to identify gene sets which are related to prostate cancer's progression using the 'molecular concept map' [9]. Their result showed state related 'molecular concepts' from normal prostate tissues to PIN (Prostate Intraepithelial Neoplasia), PCA (Prostate cancer), and metastasis. Edelman *et al *[10] used GSEA [11] analysis with 71 prostate samples consist of 22 benign, 32 PCA, and 17 metastatic tissues. They proposed several gene sets which are significantly changed in the step of n → p (normal to prostate cancer), and p → m (prostate cancer to metastasis). In these genetic level studies, researchers succeeded in clearly representing metastasis related gene sets or pathways, and in validating their results with classification tests.

As returning to the nature of metastasis biology, however, two substantial questions are emerging especially on the sample comparison step. First, do the metastasis samples really have metastasis characteristics? In Wang's work, the samples in the metastasis class are not actually metastasis samples; they are primary tumor samples which later turn out to show bad prognoses. Usually in other work, the samples used for representing metastasis are tumor samples from the very organ where the primary tumor occurred. The only difference is that the patients where the metastasis samples are from had metastatic tumors in their other organs. It is seriously doubtful whether the sample of a part of primary tumor has metastatic abilities; maybe cells with metastatic abilities already moved out to other organs, and only the other cells without the abilities have remained. Second, have other metastasis independent features been eliminated in the comparison between two samples from two distinct organs? In the case of comparing a sample from a primary tumor in an organ with another sample from metastasis tumor in another organ, there should be several elements that affect the result of the comparisons, such as tissue specificity, tissues' environmental viability, and a subtype of cancer. It is hardly expected that a result gene set represents metastatic characteristics only; large parts of the gene set might have been selected for another reasons.

In this paper, we present how to alleviate the noise effects and the lack of information in metastasis gene finding procedures using multiple and controlled analyses. We used a large scale expression profile database with rich clinical information – expO [12] (expression project for Oncology). With the clinical information, samples are categorized into several distinct sets. We investigated each set and tagged it with its intrinsic characteristics – metastatic ability, tissue specificity, and organ dependent viability. Any two combinatorial sets can be chosen for further comparisons, and the result would represent various information depends on the differences of the selected sets' characteristics.

## Methods

We describe the data sets and scoring functions in this section. First, the expression database and preprocess procedures are explained. Second, methods for converting the probe level expression values to gene and context scores are described.

### Data preparation

#### Expression profile database

The expO (expression project for Oncology) database is an archive of tumor samples with detailed clinical information. The IGC (International Genomics Consortium) has established a uniform system for obtaining and processing tissue samples for molecular characterization studies. Currently the expO database obtained 1911 tumor samples (2008-07-14) and provides the expression data through the NCBI's public gene expression database [13] (GEO – GSE2109 series). There are lots of clinical attributes associated with gene expression data such as patient's age, gender, ethnic background, tobacco use, alcohol consumption and familial history of cancer. Furthermore, cancer specific information including pathological and clinical TNM stages, cancer grades, primary tumor sites and relapse information is also available. This standardization of both microarray platform and clinical description greatly contributed to users conveniences.

### Database construction

**Figure 1:**
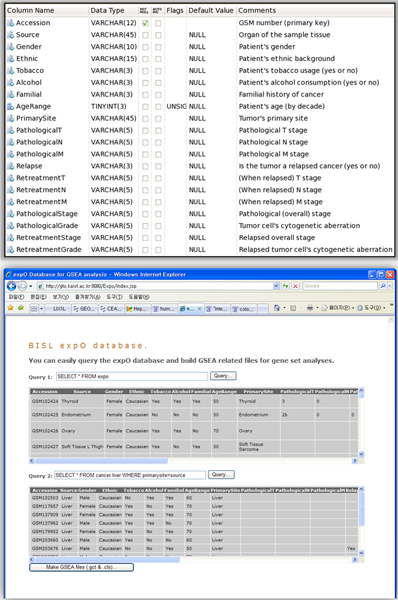
**Relational and Online Database for expO**. The flat file expO database was parsed and updated into MySQL based relational database. Database schema is shown in the upper figure. Online access to the database (lower figure) is available on

Although the expO database provides a lot of useful information with great level of standardization, the large size and the text based format (SOFT format) make it less convenient to analyze the data freely. To settle these problems, we constructed a relational database using the expO contents (Figure 1, up). Firstly, we extracted clinical information from SOFT formatted flat files using parsers. This information was uploaded into a data table (MySQL 5.0, Red-Hat Linux platform). The data table part was kept in a separated storage divided into a single GSM entry. Secondly, we constructed a web-based database (developed with JSP and JSTL) in which a user can fetch required data using SQL query statements (Figure 1, down). Finally, we built a program for automatic generation of input files used in GSEA analyses (GCT and CLS files).

### Experimental design

#### Class definition

**Figure 2:**
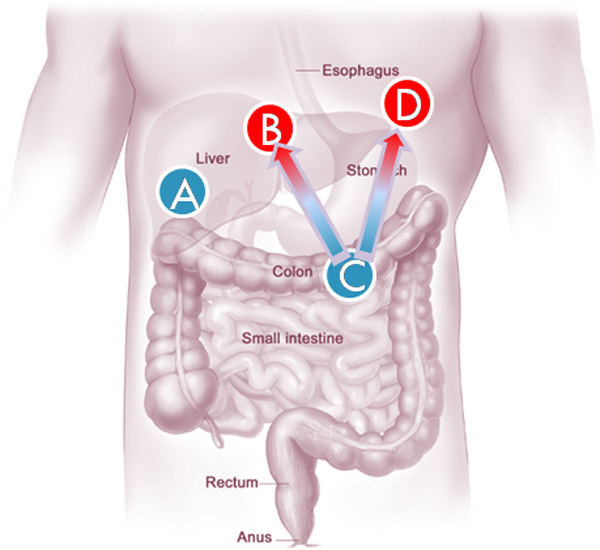
**Tumor class diagram**. Each of the four classes is described in this figure. A is a primary tumor arisen in liver. C is a primary tumor arisen in colon. B and D are both metastatic tumors disseminated from primary colon tumors. Primary tumors are denoted by blue circles, metastatic tumors are denoted by red circles.

Using the relational database and the GSEA analysis preparation program, we set up four data classes of different organ and metastatic abilities. The main concept of the analysis describes colon cancer's metastasis to liver. The organs, colon and liver, were selected due to the relative sufficiency of sample numbers than those of the other organs. The four classes are named A (a primary tumor in liver), B (metastasis tumor in liver from a primary colon tumor), C (a primary tumor in colon) and D (a metastasis tumor in other organs but liver from a primary colon tumor) respectively. Corresponding locations of four tumor classes were depicted in Figure 2. For entry details with clinical information, see Additional file 1.

#### Class A

13 samples have been assigned to class A using the below query from expO relational database.

SELECT * FROM expo WHERE source = 'liver' AND primarysite = 'liver' AND histology NOT LIKE '%metastatic%'

From the query result, the GSM203676 sample was removed as it turned out to be a relapsed cancer. Finally, class A has 12 primary liver tumors without any relapse and metastasis and collected from liver.

#### Class B

20 samples have been assigned to class B using the below query.

SELECT * FROM expo WHERE source = 'liver' AND primarysite = 'colon' AND histology LIKE '%metastatic%'

The histology phrase ensures that the primary colon tumor has metastasized to liver. Without the phrase, the secondary liver cancer might be thought to be another independent primary liver tumor after the primary colon tumor occurred.

#### Class C

188 samples have been assigned to class C using the below query.

SELECT * FROM expo WHERE source = 'colon' AND primarysite = 'colon' AND histology NOT LIKE '%metastatic%' AND pathologicalM = '0'

The pathologicalM field denotes the doctor's decision about the tumor's metastatic aspect.

#### Class D

14 highly heterogeneous samples have been assigned to the class D. We extracted 16 non-liver metastatic tumors that are originated from colon.

SELECT * from expo WHERE primarysite = 'colon' AND source ! = 'liver' AND source ! = 'colon' AND histology LIKE '%metastatic'

From the 16 query results, two samples (GSM102484, GSM137952) were removed due to the adjacency of the metastasized organs (small intestine and peritoneum). The remaining samples' secondary tumor sites included ovary, lung and bladder.

Class definitionCharacteristics of each tumor class were assessed in four criteria – metastatic ability, colon tissue specificity, liver tissue specificity and viability in liver environment. For example, class A has no metastatic ability and no colon tissue specificity but it has liver tissue specificity and liver's environmental viability, whereas class B has metastatic ability, colon tissue specificity (tumor has originated from colon cells) and viability in liver's environment but no liver tissue specificity.

Differently expressed genes from two distinct classes represent characteristic gaps between those classes (see Table 1). When we compare the class A with B, the result genes are expected to contain three kinds of characteristic differences – metastatic ability (from B), colon tissue specificity (from B) and liver tissue specificity (from A). So, if a gene α was up-regulated in class B, we expect that the gene α plays a role in metastasis or colon tissue related activities.

**Table 1:** Comparison combinations of classes and expected characteristic differences.

**Comparison**	**Tissue specificity**	**Metastatic ability**	**Environmental Viability**
**A – B**	**O **(liver VS. colon)	**O **(only in B)	**X**
**A – C**	**O **(liver VS. colon)	**X **(neither)	**O **(liver only VS. colon only)
**A – D**	**O **(liver VS. colon)	**O **(only in D)	**O **(liver only VS. anywhere but liver)
**B – C**	**X**	**O **(only in B)	**O **(colon and liver VS. colon only)
**B – D**	**X**	**X **(both)	**O **(colon and liver VS. anywhere but liver)
**C – D**	**X**	**O **(only in D)	**O **(colon only VS. anywhere but liver)

'O' is marked when a certain comparison is expected to show differences in a specific context. 'X' is marked when there is no difference.

### Scoring and analysis

Differently expressed scores have been calculated based on a t-test. A score s^i^_AB_, gene *i*'s enrichment in class A compared with class B can be obtained from the below equation.

$$S_{AB}^{i}=\frac{\mu_{A}^{i}-\mu_{B}^{i}}{\sqrt{\frac{\sigma_{A}^{i^{2}}}{n_{A}}+\frac{\sigma_{B}^{i^{2}}}{n_{B}}}}$$

where μ is the mean, n is the number of samples, and σ is the standard deviation. If the s^i^_AB _score is bigger than 0, the gene *i *is up-regulated in class A.

Gathering class combinations with a consistent context gives an overview of specific characteristics. We named the series of class combinations a context vector. For example, a metastasis context vector of gene *i *is defined as below.

**vm**^*i *^= (*s*^i^_*BA*_, *s*^i^_*BC*_, *s*^i^_*DA*_, *s*^i^_*DC*_)

where s^i^_BA _= -s^i^_AB_, s^i^_DA _= -s^i^_AD_, s^i^_DC _= -s^i^_CD_.

Each element of the metastasis context vector denotes how far a gene *i *was up-regulated in the metastatic tumor sample in contrast to another primary tumor sample. The bigger the each element's value, the higher dependency on metastasis the context vector indicates. Likewise, we can define other three context vectors – colon tissue context vector, liver tissue context vector and liver viability context vector.

$$\begin{array}{l} vc^{i}=(s_{BA}^{i},s_{CA}^{i},s_{DA}^{i})\\ vl^{i}=(s_{AB}^{i},s_{AC}^{i},s_{AD}^{i})\\ vv^{i}=(s_{AC}^{i},s_{AD}^{i},s_{BC}^{i},s_{BD}^{i}) \end{array}$$

Because we cannot jump to a conclusion that each element belongs to a specific characteristic, we need to justify the consistency of the element's directionality. For example, s^i^_AB _is used in three context values (as an s^i^_BA _form in **vm**^i ^and **vl**^i^).

A high score of s^i^_AB _can be explained in one of the three hypotheses below;

i. Gene i is down-regulated in metastatic tumors

ii. Gene i is down-regulated in colon tissues

iii. Gene i is up-regulated in liver tissues

Now, we check whether the gene *i *has been up or down-regulated in other class combinations. If the s^i^_AC_, and s^i^_AD _score were also high, all of the elements in the liver viability context vector vl^i ^have plus values indicating that the hypothesis iii – gene *i *would be up-regulated in liver tissues – would be correct. We define the consistency factor c.

$${\text{c}}_{vi}=\{\begin{array}{ll} +1 & \left({\text{every element of v}}^{\text{i}}>0\right)\\ 0 & \left({\text{v}}^{\text{i}}\text{ has both signs}\right)\\ -1 & \left({\text{every element of v}}^{\text{i}}<0\right) \end{array}$$

The final score of gene i's dependency on a specific characteristic τ is,

$$f(i,\tau )-c_{v\tau^{i}}\prod_{x}\left|v\tau_{x}^{i}\right|$$

where **vτ**^i ^is a context vector of gene i on the characteristic τ, c_vτ _i is a consistency factor of the vector **vτ**^i^.

## Results and discussion

**Figure 3:**
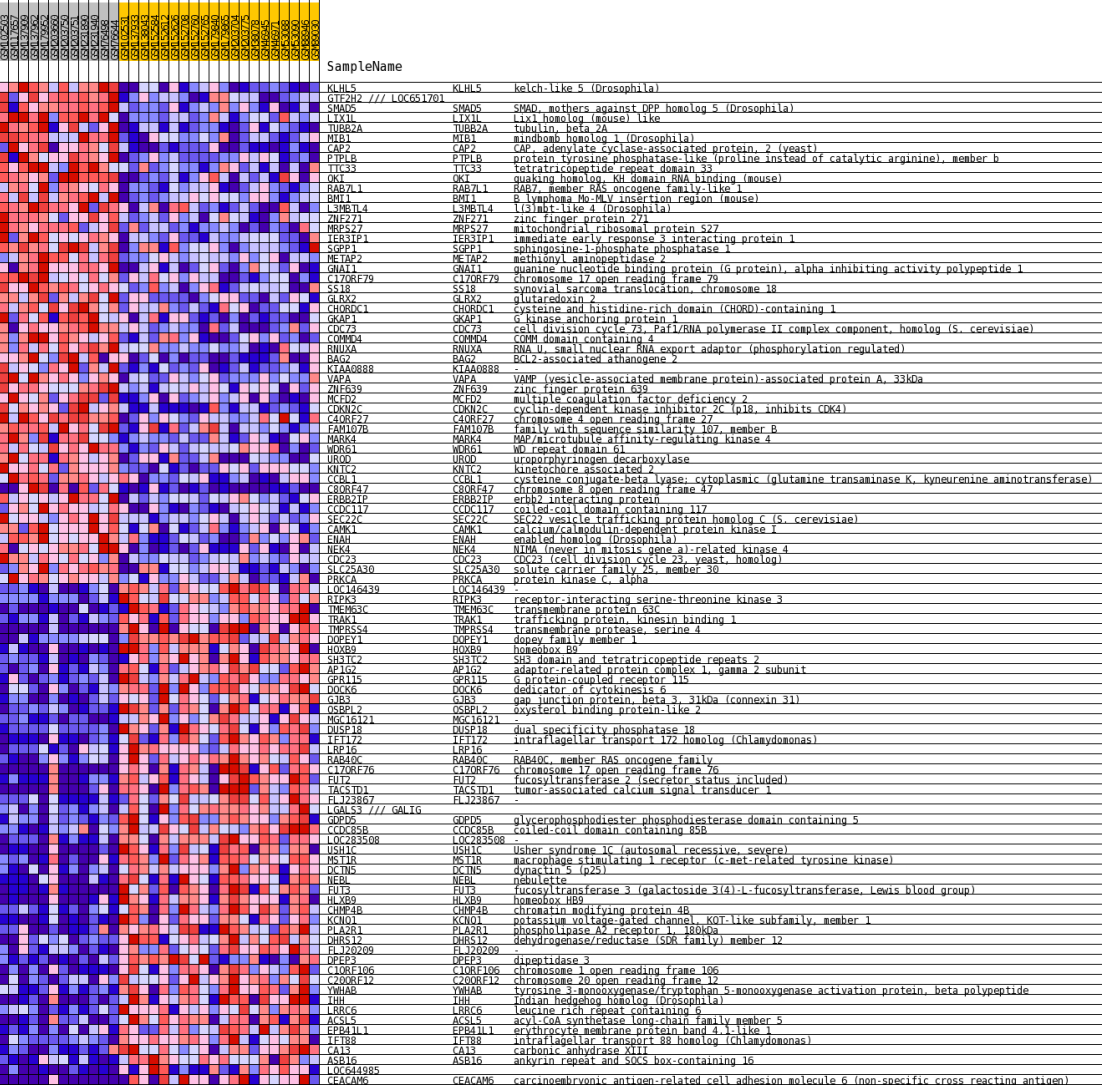
**Heat map of A↔B comparison**. Differently expressed genes were denoted in a heat map. Sample A (left cluster) is from primary liver tissues, sample B (right cluster) is from liver metastasis of colon cancer. As shown in Table 1, this result contains information of metastatic ability and tissues specificity (liver versus colon tissue). All heat maps from other comparison combinations are included in Additional file 2.

We scored 20606 genes using the function described in the last section. Total 54675 probe sets from an Affymetrix U133 Plus 2.0 chip were matched to their corresponding genes using GSEA v2 program's Collapse Dataset tool. In the case of many to gene matching, we used the maximum value of the probes. Enrichment scores have been calculated for six comparisons (A↔B, A↔C, A↔D, B↔C, B↔D, C↔D), and their differently expressed genes were denoted using six Heat Maps. The heat map of A↔B is shown in Figure 3. Remaining heat maps are shown in Additional file 2.

For each gene, the scores for dependency on a specific characteristic, f(i, **τ**) were calculated for four **τ**s – characteristics. For example, the scores of gene HPN (hepsin, transmembrane protease) with respect to the four characteristics (metastasis, colon tissue, liver tissue, and liver viability) were 0, -100.0, 100.0, and 2179 respectively, which can be interpreted like the HPN gene is not related to metastasis, but it is closely related to the cellular viability in liver as Klezovitch *et al *has reported [14]. Top genes for each characteristic were identified. We will discuss the top genes and their reliability case by case.

### Colon and liver tissue

As described in the section on Experimental design, the context vector for colon is exactly the minus signed liver context vector, because any tissues used for the sample data were colon tissues or liver tissues, exclusively. 6691 genes from total 20606 genes scored zero (their context vectors contain both signs of elements) in these characteristics. RIPK3 (receptor-interacting serine-threonine kinase 3) scored highest by 1674.1 and KLHL (kelch-like 5) gene scored lowest by -1610.9. Scores were re-scaled for more convenient readability (multiplied power of 10s depend on the size of context vectors). Mean score was 11.27 with standard deviation 85.4. Top 10 genes for all characteristics are shown in Table 2. We used the TiGER database (Tissue-specific Gene Expression and Regulation) [15] to validate the result genes' tissue specificity. In colon's case, top 10 genes were all highly regulated in colon compared to liver. TMPRSS4 (1442.7 colon score, 4^th ^ranked) and HLXB9 (Homo sapiens homeobox HB9, colon score 1125.7, 8^th ^ranked) were registered to the TiGER colon specific gene list. But in liver's case, no gene in the top 10 liver score list was registered to the database.

**Table 2:** Top 10 genes of four characteristics.

***Characteristic***	***Gene***	***Score***	***Description***
Colon tissue	RIPK3	1674.06	Receptor-interacting serine-threonine kinase 3
	LOC146439	1664.03	-
	FUT2	1463.07	Fucosyltransferase 2 (secretor status included)
	TMPRSS4	1442.70	Transmembrane protease, serine 4
	GJB3	1320.09	Gap junction protein, beta 3, 31 kDa (connexin 31)
	C1ORF106	1248.81	Chromosome 1 open reading frame 106
	USH1C	1174.67	Usher syndrome 1C (autosomal recessive, severe)
	HLXB9	1125.69	Homeobox HB9
	IFT172	1114.38	Intraflagellar transport 172 homolog (Chlamydomonas)
	DLG3	1102.68	Discs, large homolog 3 (neuroendocrine-dlg, Drosophila)

Liver tissue	KHLH5	1610.92	Kelch-like 5
	TUBB2A	1185.24	Tubulin, beta 2A
	QKI	1100.99	Quaking homolog, KH domain RNA binding
	FAM107B	798.17	Family with sequence similarity 107, member B
	RAB7L1	765.74	RAB7, member RAS oncogene family-like 1
	GTF2H2	716.89	-
	CAP2	708.44	CAP, adenylate cyclase-associated protein 2
	IL6ST	706.20	Interleukin 6 signal transducer
	PTPLB	702.20	Protein tyrosin phosphatase-like B
	CPEB4	635.24	Cytoplasmic polyadenylation elemenet binding protein

Liver viability	C4A	6614.23	Complement component 4A (Rodgers blood group)
	ALB	6216.49	Albumin
	FGG	6096.9	Fibrinogen gamma chain
	SERPINA3	5958.13	Serpin peptidase inhibitor, clade A
	FGA	5641.41	Fibrinogen alpha chain
	HP	5634.32	Haptoglobin
	LAMP2	5549.98	Lysosomal-associated membrane protein2
	FGB	5446.63	Fibrinogen beta chain
	AGT	5353.17	Angiotensinogen
	APOA2	5343.06	Apolipoprotein A-II

Metastasis	MGC16121	2032.63	-
	PLA2R1	1405.16	Phospholipase A2 receptor1, 180 kDa
	TREM2	812.81	Triggering receptor expressed on myeloid cells 2
	HS3ST2	647.47	Heparin sulfate 3-O-sulfotransferase 2
	ST6GAL2	627.44	ST6 beta-galactosamide alpha-2,6-sialytransferase 2
	ATP10A	584.84	ATPase, class V, type 10A
	BGN	563.69	Biglycan
	RAB11FIP3	546.31	RAB11 family interacting protein 3
	RAD51L1	510.23	RAD51-like 1
	C20ORF12	483.49	Chromosome 20 open reading frame 12

All genes are ranked by the final characteristic score. Functionally unknown genes or hypothetical genes are denoted by '-' in their description columns.

We concluded that extracted colon/liver tissue specific genes do not contain any universal tissue specific genes. Instead, the result includes genes relatively up or down-regulated than in the other tissues. But this result is enough to be used to offset any bias caused by the tissue differences.

### Liver viability

The same analysis was applied to the liver viability characteristics. Mean score was 6.9 with standard deviation 513.4. Surprisingly, 9 of 10 top genes were all registered to the TiGER's liver specific gene database (see Table 2). Including ALB (albumin, 6216 liver viability score, 2^nd ^ranked), FGG (fibrinogen gamma chain, 6097 liver viability score, 3^rd ^ranked) and SERPINA3 (serpin peptidase inhibitor, 5958 liver viability score, 4^th ^ranked), all the top genes were well known as liver specific genes. Despite FGG and HP/HPR (Homo sapiens haptoglobin/haptoglobin-related protein) are significantly up-regulated in the tissues from liver environment, their liver score were all zero. In the case of FGG, the DEG score from A↔B was -0.0058 making the final score 0. Similarly, HP/HPR's zero score was due to the minus DEG score in A↔B.

The differences of two liver-related scores need to be examined. One seeks to find any signs coming from liver tissue's characteristics, while the other from liver's environments. Because the liver context vector and liver viability context vector shares two elements, S^i^_AC _and S^i^_AD_, we could pay attention to only S^i^_AB_, S^i^_BC_, and S^i^_BD _elements. In the result, we found S^i^_AB _hardly catches liver specific genes. Even though the B sample came from colon tissues, we could see its expression pattern simulated that of liver tissues. Cancer cells go through increased genetic and epigenetic mutations, and sometimes their genetic instability helps by providing variety and perpetuity to themselves. During the metastasis procedures, colon cancer cells that acquired invasion and metastasis abilities possibly acquire activation of liver specific genes before or after they form micrometastasis. The tissue specific gene list of TiGER database has been established using EST (Expressed Sequence Tags) tags from sample tissues. So, the genes in the list can be identified properly not by the tissue originality but by the activity of core genes, which enables the cell to live in a liver. It is well shown in the score S^i^_BC_; even though B and C are both from colon cells, their liver specific genes are mostly up-regulated in B. In the mean time, we cannot stop concerning the possibility that all the samples collected from liver tissues contain surrounding normal liver tissues, making the entire results more ambiguous.

### Metastasis

The result shows top 10 up and down-regulated genes in metastasis samples (Table 3, down-regulated genes are not shown). Mean score was 5.45 with standard deviation 46.87. Unfortunately, any biological processes of top scored gene MGC16121 (hypothetical gene, 2032 metastasis score, 1^st ^ranked) were not discovered. PLA2R1 (phospholipase A2 receptor 1, 1405 metastasis score, 2^nd ^ranked) is known to acts as a receptor for phospholipase sPLA2-IB and also bind to snake PA2-like toxins. Binding of sPLA2-IB induces various effects [16] including activation of MAPK cascade to induce cell proliferation and inflammatory reactions which are well known metastasis procedures by increasing cellular motility and angiogenesis [17,18]. TREM2 (triggering receptor expressed on myeloid cells 2, 1405 metastasis score, 3^rd ^ranked) is also known to have a role in chronic inflammations and stimulate production of constitutive chemokines and cytokines [19]. On the other hands, PTPLB (protein tyrosine phosphatase-like member b, -1189 metastasis score, top down-regulated) is significantly down-regulated in all metastasis samples. The main function of PTPLB is not well discovered so that the direct relation to metastasis is hard to find. But PTPLB is known to interact with BAP31 [20] which is involved in CASP8-mediated apoptosis [21] which is an important pathway in tumorigenesis and metastasis resistance [22].

**Table 3:** B↔C comparison and modified result.

***Class***	***Gene***	***B vs. C***	***Liver viability***	***Difference***	***Liver and metastasis dependency***
B↔C only	COLEC11	3.95	2.45	1.49	Liver up-regulated
	NRBP2	3.29	3.57	-0.28	Liver up-regulated
	FGG	3.26	3.81	-0.55	Liver specific
	ART4	3.19	2.08	1.10	Liver specific
	LBP	3.15	3.44	-0.28	-
	GADD45B	3.15	2.82	0.33	-
	HP /// HPR	3.11	3.69	-0.58	Liver specific
	ALB	3.088	3.80	-0.71	Liver specific
	C10ORF11	3.06	2.25	0.80	-
	GCKR	3.03	3.40	-0.36	-

Modified with liver viability	MGC16121	2.43	-1.39	3.83	Unknown
	HS3ST2	2.34	-0.39	2.74	-
	DHRS12	2.11	-0.43	2.54	-
	MSR1	2.07	-0.33	2.41	Inflammation
	DNASE1	2.10	-0.23	2.33	-
	MARCO	2.86	0.55	2.31	Macrophage receptor
	SPAG4	2.18	-0.10	2.28	-
	INMT	2.93	0.71	2.22	-
	AOAH	2.45	0.26	2.19	Metastasis related
	EPO	2.20	0.14	2.05	Cancer and metastasis

The result of a naive comparison between a metastatic tumor (B) and a primary tumor (C) is shown in the upper row. This comparison is expected to represent not only metastatic ability but also liver environmental viability. Normalizing with the liver environmental viability related genes successfully eliminated many genes not selected for metastatic characteristics in the result of our methods (lower row).

To prove the enhancement of the result we compared two gene lists from B↔C and post-processed B↔C with our method. B↔C (liver metastasis of colon cancer versus primary colon cancer) is a commonly used comparison for extracting metastatic signatures. As shown in the Table 3, a result from B↔C also contains liver viability characteristics as well as metastasis characteristics. So we normalized both of the B↔C and liver viability scores and found gene sets whose B↔C scores are high but liver viability scores are low. Because the liver viability context vector already contains S^i^_BC_, we modified the context vector removing the element. As we expected, the Pearson correlation of two scores was 0.469 indicating that large parts of B↔C comparison result is due to the liver viability characteristics. As shown in Table 3, almost half of the top scored genes merely from the B↔C comparison were liver specific genes. But there was no liver specific gene in the top 20 gene list in modified results. All the top 10 genes including COLEC11 (collectin sub-family member 11), FGG (fibrinogen gamma chain), ART4 (ADP-ribosyltransferase 4), ALB (albumin), and HP (haptoglobin) were removed in the modified result. Instead, metastasis candidate genes including AOAH [23] (acyloxyacyl hydrolase), EPO [24] (erythropoietin) and MAR1 (macrophage scavenger receptor1, involved in inflammation pathways) were newly included. We are expecting the other genes would be validated further.

## Conclusion

We suggested a new method for identifying metastasis related genes from a large scale database. The proposed method attempts to minimize the influences from other factors but metastasis including tissue originality and tissue viability by confining the result of metastasis unrelated test combinations. We presented tissue specific and tissue viability related genes, and validated them using tissue specificity database, TiGER. Finally, we presented metastasis candidate genes by calculating differences of metastasis and liver viability normalized scores. We would like to expand the experiments to other tissues using remaining records of the databases and further validate the result by constructing classifiers.

## Competing interests

The authors declare that they have no competing interests.

## Authors' contributions

SK developed the fundamental idea of the work, performed experiments, validated the results, and wrote the manuscript. DL evaluated and revised the idea, and supervised manuscript processes.

## Supplementary Material

Additional file 1 Entries of four tumor classes with their clinical information Click here for file

Additional file 2 Six heat maps of inter-tumor class comparisons Click here for file
