# Supplementary material for: Mining metastasis related genes by primary-secondary tumor comparisons from large-scale databases
Source: BMC Bioinformatics. 2009 Mar 19;10(Suppl 3):S2. doi: 10.1186/1471-2105-10-S3-S2 (PMC2665050; doi:10.1186/1471-2105-10-S3-S2)
Supplement: Additional file 1 — Entries of four tumor classes with their clinical information [file 1471-2105-10-S3-S2-S1.pdf]

Supplementary Data 1. Entries of four tumor classes with their clinical information

A. Primary liver cancer

AC	Src	Gender	Ethnic	TBC	Alc	FM	Age	PSite	PT	PN	PM	RL	RT	RN	RM	P Stage	P Grade	R Stage	R Grade	Histology	CT	CN	CM
GSM102503	Liver	Male	Caucasian	No	Yes	Yes	60	Liver	-	-	-	-	-	-	-	-	-	-	-	-	-	-	-
GSM117657	Liver	Female	Caucasian	Yes	No	Yes	70	Liver	-	-	-	-	-	-	-	-	-	-	-	-	-	-	-
GSM137909	Liver	Female	Caucasian	Yes	Yes	Yes	70	Liver	-	-	-	-	-	-	-	-	-	-	-	-	-	-	-
GSM137962	Liver	Male	Caucasian	Yes	Yes	No	70	Liver	-	-	-	-	-	-	-	-	-	-	-	-	-	-	-
GSM179952	Liver	Female	Caucasian	Yes	No	Yes	70	Liver	-	-	-	-	-	-	-	-	-	-	-	-	-	-	-
GSM203660	Liver	Male	Caucasian	Yes	No	No	60	Liver	-	-	-	-	-	-	-	-	-	-	-	-	-	-	
GSM203750	Liver	Male	Caucasian	No	No	Yes	70	Liver	-	-	-	-	-	-	-	-	-	-	-	-	-	-	-
GSM203751	Liver	Male	Caucasian	No	No	Yes	60	Liver	-	-	-	-	-	-	-	-	-	-	-	-	-	-	-
GSM231890	Liver	Female	Caucasian	Yes	No	No	50	Liver	-	-	-	-	-	-	-	-	-	-	-	-	-	-	-
GSM231940	Liver	Female	Caucasian	No	No	Yes	60	Liver	-	-	-	-	-	-	-	-	-	-	-	-	-	-	-
GSM76498	Liver	Female	Caucasian	No	No	No	70	Liver	-	-	-	-	-	-	-	-	-	-	-	-	-	-	-
GSM76644	Liver	Male	Caucasian	Yes	No	No	70	Liver	-	-	-	-	-	-	-	-	-	-	-	-	-	-	-

AC: Accession number

Src: Source. An organ or location of sample source for microarray experiment

TBC: Tobacco use (Yes/No)

Alc: Alcohol consumption (Yes/No)

FM: Familial history of cancer (Yes/No)

Age: Age range of the patients in decades

PSite: Primary site of the cancer

PT, PN, PM: Pathological T, N, M

RL: Relapsed tumor (Yes/No)

RT, RN, RM: Retreatment T, N, M

PStage, PGrade, RStage, RGrade: Pathological stage, Pathological grade, Retreatment stage, Retreatment grade

CT, CN, CM: Clinical T, N, M

B. Metastatic liver cancer from primary colon cancer

AC	Src	Gender	Ethnic	TBC	Alc	FM	Age	P Site	P T	P N	P M	R L	R T	R N	R M	P Stage	P Grade	R Stage	R Grade	Histology	C T	C N	C M
GSM102531	Liver	Male	Caucasian	No	No	Yes	70	Colon	-	-	-	Yes	3	2	1	-	-	-	-	Metastatic Adenocarcinoma	-	-	-
GSM137933	Liver	Male	Caucasian	No	No	Yes	50	Colon	-	-	-	Yes	3	1	1	-	-	-	-	Metastatic Adenocarcinoma	-	-	-
GSM138043	Liver	Male	Caucasian	Yes	No	No	50	Colon	-	-	-	Yes	3	0	1	-	-	-	-	Metastatic Adenocarcinoma	-	-	-
GSM152584	Liver	Male	Caucasian	Yes	Yes	No	60	Colon	-	-	-	-	-	-	-	-	-	-	-	Metastatic Adenocarcinoma	X	X	1
GSM152612	Liver	Male	Caucasian	Yes	Yes	Yes	60	Colon	-	-	-	Yes	X	X	1	-	-	-	-	Metastatic Mucinous Carcinoma	-	-	-
GSM152626	Liver	Male	Caucasian	No	No	Yes	60	Colon	-	-	-	Yes	3	0	1	-	-	-	-	Metastatic Adenocarcinoma	-	-	-
GSM152708	Liver	Female	Caucasian	No	No	No	40	Colon	-	-	-	Yes	2	1	1	-	-	-	-	Metastatic Adenocarcinoma	-	-	-
GSM152760	Liver	Male	Caucasian	Yes	No	Yes	50	Colon	-	-	-	Yes	X	X	1	-	-	-	-	Metastatic Adenocarcinoma	-	-	-
GSM152765	Liver	Female	Caucasian	No	No	No	80	Colon	-	-	-	Yes	3	1	1	-	-	-	-	Metastatic Adenocarcinoma	-	-	-
GSM179840	Liver	Male	Caucasian	No	No	Yes	60	Colon	-	-	-	Yes	1	1	1	-	-	-	-	Metastatic Adenocarcinoma	-	-	-
GSM179865	Liver	Female	Caucasian	Yes	No	Yes	40	Colon	-	-	-	Yes	X	X	1	-	-	-	-	Metastatic Adenocarcinoma	-	-	-
GSM203704	Liver	Male	Caucasian	Yes	No	No	70	Colon	-	-	-	Yes	X	X	1	-	-	-	-	Metastatic Adenocarcinoma	-	-	-
GSM203775	Liver	Male	Caucasian	Yes	No	Yes	70	Colon	4	1	1	-	-	-	-	4	2	-	-	Metastatic Adenocarcinoma	-	-	-
GSM38078	Liver	Female	Caucasian	No	No	Yes	60	Colon	4	2	1	-	-	-	-	4	3	-	-	Metastatic Adenocarcinoma	-	-	-
GSM46945	Liver	Female	Caucasian	Yes	No	Yes	40	Colon	3	0	1	-	-	-	-	4	2	-	-	Metastatic Adenocarcinoma	-	-	-
GSM46971	Liver	Female	Caucasian	Yes	No	Yes	40	Colon	4	0	1	-	-	-	-	4	2	-	-	Metastatic Adenocarcinoma	-	-	-
GSM53088	Liver	Female	Caucasian	Yes	No	Yes	60	Colon	-	-	-	Yes	-	-	-	-	-	-	-	Metastatic Mucinous Carcinoma	-	-	-
GSM53090	Liver	Male	Caucasian	Yes	Yes	No	60	Colon	-	-	-	Yes	-	-	-	-	-	-	-	Metastatic Mucinous Carcinoma	-	-	-
GSM88946	Liver	Male	Caucasian	No	No	Yes	40	Colon	X	1	1	-	-	-	-	4	2	-	-	Metastatic Adenocarcinoma	-	-	-
GSM89030	Liver	Male	Caucasian	No	No	Yes	60	Colon	-	-	-	Yes	X	X	1	-	-	-	-	Metastatic Adenocarcinoma	-	-	-

C. Primary colon cancer

AC	Src	Gender	Ethnic	TBC	Alc	FM	Age	PSite	P T	P N	P M	R L	R T	R N	R M	P Stage	P Grade	R Stage	R Grade	Histology	C T	C N	C M
GSM102429	Colon	Male	Caucasian	No	No	No	70	Colon	3	0	0	-	-	-	-	2A	2	-	-	Adenocarcinoma	-	-	-
GSM102431	Colon	Male	Caucasian	No	No	No	70	Colon	2	1	0	-	-	-	-	3A	2	-	-	Adenocarcinoma	-	-	-
GSM102436	Colon	Female	Caucasian	No	No	No	70	Colon	4	2	0	-	-	-	-	3C	2	-	-	Mucinous Carcinoma	-	-	-
GSM102460	Colon	Male	Caucasian	No	Yes	Yes	50	Colon	3	0	0	-	-	-	-	2A	2	-	-	Adenocarcinoma	-	-	-
GSM102485	Colon	Male	Caucasian	Yes	No	Yes	70	Colon	3	0	0	-	-	-	-	2A	3	-	-	Adenocarcinoma	-	-	-
GSM102497	Colon	Male	Caucasian	No	No	Yes	50	Colon	1	1	0	-	-	-	-	3A	3	-	-	Adenocarcinoma	-	-	-
GSM102501	Colon	Female	Caucasian	No	Yes	Yes	60	Colon	3	2	0	-	-	-	-	3C	2	-	-	Adenocarcinoma	-	-	-
GSM102516	Colon	Male	Caucasian	Yes	No	Yes	70	Colon	3	0	0	-	-	-	-	2A	3	-	-	Adenocarcinoma	-	-	-
GSM102518	Colon	Female	Caucasian	No	No	Yes	70	Colon	2	1	0	-	-	-	-	3A	2	-	-	Adenocarcinoma	-	-	-
GSM102519	Colon	Female	Caucasian	No	No	No	80	Colon	3	0	0	-	-	-	-	2A	2	-	-	Adenocarcinoma	-	-	-
GSM102524	Colon	Male	Caucasian	No	Yes	No	60	Colon	3	1	0	-	-	-	-	3B	2	-	-	Adenocarcinoma	-	-	-
GSM102540	Colon	Female	Caucasian	Yes	No	No	70	Colon	3	2	0	-	-	-	-	3C	3	-	-	Adenocarcinoma	-	-	-
GSM102549	Colon	Male	Caucasian	Yes	Yes	No	80	Colon	2	0	0	-	-	-	-	1	2	-	-	Mucinous Carcinoma	-	-	-
GSM102550	Colon	Female	Caucasian	No	No	Yes	80	Colon	3	0	0	-	-	-	-	2A	3	-	-	Adenocarcinoma	-	-	-
GSM102551	Colon	Female	Caucasian	No	No	No	70	Colon	3	0	0	-	-	-	-	2A	2	-	-	Mucinous Carcinoma	-	-	-
GSM102559	Colon	Male	Caucasian	Yes	Yes	Yes	50	Colon	3	0	0	-	-	-	-	2A	2	-	-	Adenocarcinoma	-	-	-
GSM102561	Colon	Male	Caucasian	Yes	Yes	Yes	70	Colon	2	0	0	-	-	-	-	1	2	-	-	Adenocarcinoma	-	-	-
GSM102572	Colon	Female	Caucasian	No	No	Yes	50	Colon	2	0	0	-	-	-	-	1	2	-	-	Adenocarcinoma	-	-	-
GSM102577	Colon	Female	American Indian	No	No	Yes	50	Colon	3	0	0	-	-	-	-	2A	2	-	-	Adenocarcinoma	-	-	-
GSM102579	Colon	Male	Caucasian	Yes	No	Yes	70	Colon	2	0	0	-	-	-	-	1	2	-	-	Mucinous Carcinoma	-	-	-
GSM102581	Colon	Male	Caucasian	No	Yes	Yes	40	Colon	4	0	0	-	-	-	-	2B	4	-	-	Adenocarcinoma	-	-	-
GSM117635	Colon	Female	Caucasian	Yes	Yes	Yes	60	Colon	3	0	0	-	-	-	-	2A	2	-	-	Adenocarcinoma	-	-	-
GSM117642	Colon	Male	African-America	No	Yes	Yes	50	Colon	3	2	0	-	-	-	-	3C	2	-	-	Adenocarcinoma	-	-	-
GSM117649	Colon	Female	Caucasian	No	No	No	70	Colon	3	0	0	-	-	-	-	2A	3	-	-	Adenocarcinoma	-	-	-
GSM117656	Colon	Male	Caucasian	Yes	No	No	60	Colon	2	0	0	-	-	-	-	1	3	-	-	Adenocarcinoma	-	-	-
GSM117662	Colon	Male	Caucasian	Yes	No	No	70	Colon	3	0	0	-	-	-	-	2A	2	-	-	Adenocarcinoma	-	-	-
GSM117664	Colon	Female	Caucasian	Yes	No	No	60	Colon	4	0	0	-	-	-	-	2B	3	-	-	Adenocarcinoma	-	-	-
GSM117672	Colon	Female	Caucasian	No	No	No	70	Colon	1	0	0	-	-	-	-	1	-	-	-	Adenocarcinoma arising in a villous adenoma	-	-	-
GSM117673	Colon	Male	Caucasian	No	No	No	50	Colon	3	1	0	-	-	-	-	3B	2	-	-	Adenocarcinoma	-	-	-
GSM117676	Colon	Male	Caucasian	Yes	No	Yes	70	Colon	1	0	0	-	-	-	-	1	2	-	-	Adenocarcinoma	-	-	-
GSM117681	Colon	Male	Caucasian	No	No	Yes	60	Colon	2	0	0	-	-	-	-	1	2	-	-	Adenocarcinoma arising in a villous adenoma	-	-	-
GSM117709	Colon	Female	Caucasian	Yes	Yes	Yes	80	Colon	4	0	0	-	-	-	-	2B	2	-	-	Adenocarcinoma	-	-	-
GSM117720	Colon	Male	African-America	Yes	Yes	No	70	Colon	2	0	0	-	-	-	-	1	2	-	-	Adenocarcinoma	-	-	-

GSM117728	Colon	Male	Caucasian	No	No	No	70	Colon	3	0	0	-	-	-	-	2A	2	-	-	Adenocarcinoma	-	-	-
GSM117738	Colon	Female	Caucasian	Yes	No	Yes	70	Colon	3	1	0	-	-	-	-	3B	2	-	-	Adenocarcinoma	-	-	-
GSM117742	Colon	Male	Caucasian	No	No	Yes	50	Colon	3	0	0	-	-	-	-	2A	2	-	-	Mucinous Carcinoma	-	-	-
GSM117746	Colon	Female	Caucasian	No	No	No	60	Colon	2	1	0	-	-	-	-	3A	2	-	-	Mucinous Carcinoma	-	-	-
GSM117747	Colon	Female	Caucasian	Yes	No	No	70	Colon	3	0	0	-	-	-	-	2A	3	-	-	Adenocarcinoma	-	-	-
GSM117752	Colon	Male	Caucasian	Yes	Yes	No	60	Colon	3	2	0	-	-	-	-	3C	2	-	-	Mucin-producing adenocarcinoma	-	-	-
GSM117775	Colon	Female	Caucasian	Yes	No	No	80	Colon	3	0	0	-	-	-	-	2A	2	-	-	Adenocarcinoma	-	-	-
GSM137922	Colon	Female	Caucasian	No	No	Yes	70	Colon	2	0	0	-	-	-	-	1	2	-	-	Adenocarcinoma arising in a villous adenoma	-	-	-
GSM137947	Colon	Male	Caucasian	Yes	No	No	70	Colon	3	0	0	-	-	-	-	2A	2	-	-	Adenocarcinoma	-	-	-
GSM137949	Colon	Female	Caucasian	No	No	No	50	Colon	3	0	0	-	-	-	-	2A	3	-	-	Adenocarcinoma	-	-	-
GSM137967	Colon	Female	Caucasian	Yes	No	No	70	Colon	3	0	0	-	-	-	-	2A	2	-	-	Adenocarcinoma	-	-	-
GSM137972	Colon	Male	Asian	No	No	Yes	70	Colon	3	0	0	-	-	-	-	2A	3	-	-	Adenocarcinoma	-	-	-
GSM137993	Colon	Male	American Indian	No	No	Yes	50	Colon	3	0	0	-	-	-	-	2A	2	-	-	Adenocarcinoma	-	-	-
GSM138005	Colon	Female	Caucasian	No	Yes	No	50	Colon	2	0	0	-	-	-	-	1	2	-	-	Adenocarcinoma	-	-	-
GSM138007	Colon	Female	Caucasian	Yes	No	No	70	Colon	3	2	0	-	-	-	-	3C	2	-	-	Adenocarcinoma	-	-	-
GSM138015	Colon	Female	Caucasian	Yes	No	Yes	50	Colon	3	0	0	-	-	-	-	2A	2	-	-	Adenocarcinoma	-	-	-
GSM138018	Colon	Female	Caucasian	Yes	Yes	Yes	50	Colon	3	1	0	-	-	-	-	3B	2	-	-	Adenocarcinoma	-	-	-
GSM138022	Colon	Male	Caucasian	Yes	No	No	70	Colon	2	0	0	-	-	-	-	1	2	-	-	Adenocarcinoma arising in a villous adenoma	-	-	-
GSM138032	Colon	Female	Caucasian	Yes	No	Yes	70	Colon	3	1	0	-	-	-	-	3B	3	-	-	Adenocarcinoma	-	-	-
GSM138037	Colon	Male	Caucasian	No	No	Yes	40	Colon	2	0	0	-	-	-	-	1	2	-	-	Adenocarcinoma	-	-	-
GSM138044	Colon	Male	Caucasian	Yes	No	Yes	70	Colon	3	0	0	-	-	-	-	2A	2	-	-	Adenocarcinoma	-	-	-
GSM138048	Colon	Male	Caucasian	No	No	Yes	60	Colon	3	0	0	-	-	-	-	2A	3	-	-	Adenocarcinoma	-	-	-
GSM138050	Colon	Male	Caucasian	Yes	Yes	No	50	Colon	3	0	0	-	-	-	-	2A	2	-	-	Mucinous Carcinoma	-	-	-
GSM138052	Colon	Female	Caucasian	No	No	Yes	70	Colon	3	0	0	-	-	-	-	2A	2	-	-	Adenocarcinoma	-	-	-
GSM152591	Colon	Female	Caucasian	No	No	No	70	Colon	3	0	0	-	-	-	-	2A	2	-	-	Adenocarcinoma	-	-	-
GSM152602	Colon	Male	Caucasian	No	No	Yes	50	Colon	2	0	0	-	-	-	-	1	2	-	-	Adenocarcinoma	-	-	-
GSM152610	Colon	Male	Caucasian	Yes	Yes	No	50	Colon	3	0	0	-	-	-	-	2A	2	-	-	Mucin-producing adenocarcinoma	-	-	-
GSM152613	Colon	Male	Caucasian	No	No	No	80	Colon	3	1	0	-	-	-	-	3B	3	-	-	Adenocarcinoma	-	-	-
GSM152614	Colon	Female	Caucasian	No	No	No	70	Colon	3	0	0	-	-	-	-	2A	2	-	-	Adenocarcinoma	-	-	-
GSM152632	Colon	Female	Caucasian	No	No	Yes	60	Colon	1	0	0	-	-	-	-	1	2	-	-	Adenocarcinoma	-	-	-
GSM152658	Colon	Female	Caucasian	No	No	No	50	Colon	3	0	0	-	-	-	-	2A	2	-	-	Adenocarcinoma	-	-	-
GSM152664	Colon	Female	Caucasian	Yes	No	Yes	60	Colon	3	2	0	-	-	-	-	3C	2	-	-	Adenocarcinoma	-	-	-
GSM152666	Colon	Male	Caucasian	No	Yes	Yes	40	Colon	2	0	0	-	-	-	-	1	2	-	-	Mucinous Carcinoma	-	-	-
GSM152684	Colon	Female	Caucasian	Yes	No	No	60	Colon	2	0	0	-	-	-	-	1	2	-	-	Adenocarcinoma	-	-	-
GSM152695	Colon	Male	Caucasian	Yes	No	No	70	Colon	3	2	0	-	-	-	-	3C	3	-	-	Adenocarcinoma	-	-	-
GSM152720	Colon	Female	Caucasian	Yes	Yes	No	50	Colon	3	0	0	-	-	-	-	2A	2	-	-	Medullary Carcinoma	-	-	-

GSM152725	Colon	Male	Caucasian	Yes	Yes	No	60	Colon	1	0	0	-	-	-	-	1	2	-	-	Adenocarcinoma	-	-	-
GSM152730	Colon	Female	African-America	No	Yes	No	40	Colon	3	2	0	-	-	-	-	3C	3	-	-	Adenocarcinoma	-	-	-
GSM152762	Colon	Male	Caucasian	No	No	Yes	80	Colon	3	0	0	-	-	-	-	2A	2	-	-	Adenocarcinoma arising in a villous adenoma	-	-	-
GSM152780	Colon	Male	Caucasian	No	Yes	No	40	Colon	3	0	0	-	-	-	-	2A	2	-	-	Adenocarcinoma	-	-	-
GSM152799	Colon	Male	Caucasian	Yes	No	No	70	Colon	3	0	0	-	-	-	-	2A	2	-	-	Adenocarcinoma	-	-	-
GSM179793	Colon	Female	Caucasian	Yes	No	No	70	Colon	2	1	0	-	-	-	-	3A	3	-	-	Adenocarcinoma	-	-	-
GSM179795	Colon	Male	Caucasian	Yes	Yes	No	70	Colon	3	2	0	-	-	-	-	3C	2	-	-	Adenocarcinoma	-	-	-
GSM179803	Colon	Male	Caucasian	Yes	Yes	Yes	30	Colon	3	0	0	-	-	-	-	2A	2	-	-	Adenocarcinoma	-	-	-
GSM179804	Colon	Male	Caucasian	Yes	No	No	70	Colon	3	0	0	-	-	-	-	2A	2	-	-	Adenocarcinoma	-	-	-
GSM179820	Colon	-	Caucasian	Yes	No	Yes	70	Colon	2	0	0	-	-	-	-	1	2	-	-	Adenocarcinoma	-	-	-
GSM179831	Colon	Male	Caucasian	Yes	Yes	Yes	60	Colon	3	2	0	-	-	-	-	3C	3	-	-	Adenocarcinoma	-	-	-
GSM179838	Colon	Male	Caucasian	Yes	Yes	Yes	60	Colon	2	0	0	-	-	-	-	1	2	-	-	Adenocarcinoma	-	-	-
GSM179839	Colon	Female	Caucasian	No	No	No	60	Colon	3	0	0	-	-	-	-	2A	2	-	-	Adenocarcinoma	-	-	-
GSM179844	Colon	Female	Caucasian	No	No	No	50	Colon	2	0	0	-	-	-	-	1	2	-	-	Adenocarcinoma	-	-	-
GSM179859	Colon	Female	Caucasian	No	Yes	Yes	70	Colon	2	0	0	-	-	-	-	1	3	-	-	Adenocarcinoma arising in a villous adenoma	-	-	-
GSM179860	Colon	Female	Caucasian	Yes	Yes	Yes	70	Colon	3	0	0	-	-	-	-	2A	2	-	-	Adenocarcinoma	-	-	-
GSM179880	Colon	Male	Caucasian	No	Yes	Yes	40	Colon	3	1	0	-	-	-	-	3B	2	-	-	Adenocarcinoma	-	-	-
GSM179882	Colon	Female	Caucasian	Yes	Yes	Yes	40	Colon	3	2	0	-	-	-	-	3C	2	-	-	Adenocarcinoma	-	-	-
GSM179887	Colon	Male	Caucasian	Yes	No	Yes	80	Colon	3	0	0	-	-	-	-	2A	2	-	-	Adenocarcinoma	-	-	-
GSM179889	Colon	Male	Caucasian	Yes	No	Yes	70	Colon	2	0	0	-	-	-	-	1	2	-	-	Adenocarcinoma	-	-	-
GSM179897	Colon	Female	Caucasian	No	No	Yes	50	Colon	3	0	0	-	-	-	-	2A	2	-	-	Adenocarcinoma	-	-	-
GSM179899	Colon	Female	Caucasian	No	No	Yes	50	Colon	3	0	0	-	-	-	-	2A	2	-	-	Adenocarcinoma	-	-	-
GSM179908	Colon	Female	Caucasian	No	No	No	60	Colon	3	1	0	-	-	-	-	3B	2	-	-	Adenocarcinoma	-	-	-
GSM179922	Colon	Male	Caucasian	Yes	Yes	No	60	Colon	3	0	0	-	-	-	-	2A	2	-	-	Adenocarcinoma	-	-	-
GSM179924	Colon	Female	Caucasian	No	No	Yes	90	Colon	4	0	0	-	-	-	-	2B	3	-	-	Adenocarcinoma	-	-	-
GSM179930	Colon	Female	Caucasian	Yes	Yes	No	70	Colon	2	1	0	-	-	-	-	3A	2	-	-	Adenocarcinoma	-	-	-
GSM179937	Colon	Female	Caucasian	No	Yes	Yes	70	Colon	3	1	0	-	-	-	-	3B	2	-	-	Adenocarcinoma	-	-	-
GSM203625	Colon	Female	Caucasian	Yes	Yes	Yes	70	Colon	1	0	0	-	-	-	-	1	2	-	-	Adenocarcinoma	-	-	-
GSM203627	Colon	Male	Caucasian	No	No	Yes	80	Colon	4	1	0	-	-	-	-	3B	2	-	-	Mucinous Carcinoma	-	-	-
GSM203640	Colon	Male	Caucasian	Yes	No	No	70	Colon	2	1	0	-	-	-	-	3A	3	-	-	Adenocarcinoma	-	-	-
GSM203642	Colon	Male	Caucasian	Yes	No	No	80	Colon	3	0	0	-	-	-	-	2A	2	-	-	Adenocarcinoma	-	-	-
GSM203645	Colon	Female	Caucasian	No	Yes	Yes	30	Colon	3	2	0	-	-	-	-	3C	2	-	-	Adenocarcinoma	-	-	-
GSM203653	Colon	Male	Caucasian	Yes	Yes	Yes	70	Colon	3	2	0	-	-	-	-	3C	3	-	-	Mucinous Carcinoma	-	-	-
GSM203667	Colon	Female	Caucasian	No	No	Yes	80	Colon	3	0	0	-	-	-	-	2A	2	-	-	Adenocarcinoma	-	-	-
GSM203673	Colon	Female	Caucasian	No	No	No	70	Colon	1	0	0	-	-	-	-	1	2	-	-	Adenocarcinoma arising in a villous adenoma	-	-	-
GSM203674	Colon	Male	Caucasian	No	No	No	70	Colon	3	1	0	-	-	-	-	3B	3	-	-	Adenocarcinoma	-	-	-
GSM203684	Colon	Male	Caucasian	Yes	Yes	Yes	60	Colon	3	1	0	-	-	-	-	3B	2	-	-	Adenocarcinoma	-	-	-

GSM203687	Colon	Female	Caucasian	No	Yes	No	50	Colon	2	0	0	-	-	-	-	1	2	-	-	Adenocarcinoma arising in a villous adenoma	-	-	-
GSM203700	Colon	Male	Caucasian	No	Yes	No	70	Colon	3	0	0	-	-	-	-	2A	2	-	-	Adenocarcinoma	-	-	-
GSM203702	Colon	Male	Caucasian	No	No	No	30	Colon	2	1	0	-	-	-	-	3A	3	-	-	Adenocarcinoma	-	-	-
GSM203705	Colon	Female	Caucasian	No	No	Yes	80	Colon	3	1	0	-	-	-	-	3B	2	-	-	Adenocarcinoma	-	-	-
GSM203723	Colon	Female	Caucasian	Yes	Yes	No	70	Colon	1	0	0	-	-	-	-	1	2	-	-	Adenocarcinoma arising in a villous adenoma	-	-	-
GSM203728	Colon	Male	Caucasian	Yes	No	Yes	50	Colon	3	2	0	-	-	-	-	3C	1	-	-	Adenocarcinoma	-	-	-
GSM203731	Colon	Male	Caucasian	Yes	Yes	No	70	Colon	is	0	0	-	-	-	-	0	1	-	-	Carcinoma in situ arising in a villous adenoma	-	-	-
GSM203733	Colon	Female	Caucasian	No	Yes	Yes	30	Colon	3	1	0	-	-	-	-	3B	2	-	-	Adenocarcinoma	-	-	-
GSM203755	Colon	Female	Caucasian	Yes	No	Yes	80	Colon	3	0	0	-	-	-	-	2A	2	-	-	Mucinous Carcinoma	-	-	-
GSM203782	Colon	Female	Caucasian	No	No	No	90	Colon	3	0	0	-	-	-	-	2A	3	-	-	Adenocarcinoma	-	-	-
GSM231868	Colon	Female	Caucasian	No	No	No	70	Colon	3	2	0	-	-	-	-	3C	2	-	-	Adenocarcinoma	-	-	-
GSM231875	Colon	Female	Caucasian	No	Yes	Yes	50	Colon	3	1	0	-	-	-	-	3B	2	-	-	Adenocarcinoma	-	-	-
GSM231879	Colon	Female	African-America	No	No	No	70	Colon	3	0	0	-	-	-	-	2A	2	-	-	Adenocarcinoma arising in a villous adenoma	-	-	-
GSM231905	Colon	Female	Caucasian	No	No	Yes	70	Colon	3	0	0	-	-	-	-	2A	2	-	-	Mucinous Carcinoma	-	-	-
GSM231908	Colon	Male	American Indian	Yes	No	Yes	60	Colon	3	0	0	-	-	-	-	2A	2	-	-	Mucinous Carcinoma	-	-	-
GSM231915	Colon	Female	Caucasian	No	Yes	Yes	50	Colon	3	1	0	-	-	-	-	3B	2	-	-	Adenocarcinoma arising in a villous adenoma	-	-	-
GSM231921	Colon	Female	Caucasian	No	No	Yes	50	Colon	2	0	0	-	-	-	-	1	2	-	-	Adenocarcinoma	-	-	-
GSM231928	Colon	Female	Caucasian	Yes	Yes	Yes	50	Colon	3	1	0	-	-	-	-	3B	2	-	-	Mucinous Carcinoma	-	-	-
GSM231936	Colon	Male	African-America	Yes	Yes	No	80	Colon	4	2	0	-	-	-	-	3C	3	-	-	Mucinous Carcinoma	-	-	-
GSM231955	Colon	Male	Caucasian	Yes	No	-	50	Colon	3	1	0	-	-	-	-	3B	3	-	-	Signet Ring Cell Carcinoma	-	-	-
GSM231956	Colon	Female	Caucasian	Yes	No	No	70	Colon	3	0	0	-	-	-	-	2A	2	-	-	Mucinous Carcinoma	-	-	-
GSM231958	Colon	Male	Caucasian	Yes	Yes	Yes	60	Colon	3	1	0	-	-	-	-	3B	2	-	-	Adenocarcinoma	-	-	-
GSM231961	Colon	Male	Caucasian	Yes	No	No	80	Colon	3	1	0	-	-	-	-	3B	2	-	-	Adenocarcinoma	-	-	-
GSM231971	Colon	Male	Caucasian	Yes	No	No	70	Colon	3	1	0	-	-	-	-	3B	2	-	-	Adenocarcinoma	-	-	-
GSM231988	Colon	Female	Caucasian	Yes	No	No	90	Colon	3	0	0	-	-	-	-	2A	2	-	-	Adenocarcinoma	-	-	-
GSM38055	Colon	Male	Caucasian	Yes	Yes	Yes	60	Colon	3	1	0	-	-	-	-	3B	X	-	-	Adenocarcinoma	-	-	-
GSM38074	Colon	Female	Caucasian	Yes	Yes	No	70	Colon	3	1	0	-	-	-	-	3B	2	-	-	Adenocarcinoma	-	-	-
GSM38075	Colon	Male	Caucasian	No	No	Yes	60	Colon	2	0	0	-	-	-	-	1	2	-	-	Adenocarcinoma	-	-	-
GSM38105	Colon	Female	Caucasian	Yes	No	No	50	Colon	3	1	0	-	-	-	-	3B	2	-	-	Adenocarcinoma	-	-	-
GSM38107	Colon	Female	Caucasian	Yes	No	Yes	70	Colon	3	1	0	-	-	-	-	3B	2	-	-	Adenocarcinoma	-	-	-

GSM46832	Colon	Female	Caucasian	Yes	No	No	60	Colon	is	0	0	-	-	-	-	0	-	-	-	Carcinoma in situ arising in a villous adenoma	-	-	-
GSM46841	Colon	Female	Caucasian	Yes	No	Yes	70	Colon	3	1	0	-	-	-	-	3B	3	-	-	Adenocarcinoma	-	-	-
GSM46861	Colon	Male	Caucasian	Yes	No	Yes	60	Colon	3	1	0	-	-	-	-	3B	2	-	-	Adenocarcinoma	-	-	-
GSM46864	Colon	Male	Caucasian	Yes	No	Yes	50	Colon	3	0	0	-	-	-	-	2A	2	-	-	Mucinous Carcinoma	-	-	-
GSM46878	Colon	Female	Caucasian	Yes	No	Yes	50	Colon	3	0	0	-	-	-	-	2A	2	-	-	Adenocarcinoma arising in a villous adenoma	-	-	-
GSM46887	Colon	Female	American Indian	No	No	Yes	60	Colon	2	1	0	-	-	-	-	3A	2	-	-	Adenocarcinoma	-	-	-
GSM46895	Colon	Female	Caucasian	Yes	No	Yes	80	Colon	3	0	0	-	-	-	-	2A	2	-	-	Adenocarcinoma	-	-	-
GSM46921	Colon	Female	Caucasian	No	No	No	70	Colon	3	2	0	-	-	-	-	3C	3	-	-	Mucinous Carcinoma	-	-	-
GSM46930	Colon	Female	Caucasian	Yes	No	Yes	70	Colon	3	0	0	-	-	-	-	2A	1	-	-	Adenocarcinoma	-	-	-
GSM46931	Colon	Female	Caucasian	No	Yes	Yes	40	Colon	3	1	0	-	-	-	-	3B	2	-	-	Adenocarcinoma	-	-	-
GSM53055	Colon	Male	Caucasian	Yes	Yes	No	60	Colon	2	0	0	-	-	-	-	1	3	-	-	Adenocarcinoma	-	-	-
GSM53106	Colon	Female	Caucasian	No	No	No	70	Colon	3	0	0	-	-	-	-	2A	1	-	-	Adenocarcinoma	-	-	-
GSM53113	Colon	Female	Caucasian	No	No	Yes	70	Colon	3	0	0	-	-	-	-	2A	3	-	-	Adenocarcinoma	-	-	-
GSM53126	Colon	Male	Caucasian	Yes	No	Yes	40	Colon	2	0	0	-	-	-	-	1	2	-	-	Adenocarcinoma	-	-	-
GSM53178	Colon	Male	Caucasian	Yes	Yes	Yes	50	Colon	3	0	0	-	-	-	-	2A	2	-	-	Adenocarcinoma	-	-	-
GSM76501	Colon	Male	Caucasian	No	No	No	70	Colon	3	1	0	-	-	-	-	3B	2	-	-	Adenocarcinoma	-	-	-
GSM76519	Colon	Male	Caucasian	No	Yes	No	50	Colon	3	1	0	-	-	-	-	3B	3	-	-	Adenocarcinoma	-	-	-
GSM76520	Colon	Male	Caucasian	Yes	Yes	No	70	Colon	3	0	0	-	-	-	-	2A	4	-	-	Adenocarcinoma	-	-	-
GSM76522	Colon	Male	American Indian	Yes	Yes	Yes	70	Colon	3	0	0	-	-	-	-	2A	2	-	-	Adenocarcinoma	-	-	-
GSM76524	Colon	Female	Caucasian	Yes	No	Yes	70	Colon	3	0	0	-	-	-	-	2A	2	-	-	Adenocarcinoma	-	-	-
GSM76526	Colon	Female	Caucasian	No	Yes	No	60	Colon	3	1	0	-	-	-	-	3B	2	-	-	Adenocarcinoma	-	-	-
GSM76548	Colon	Female	Caucasian	Yes	No	No	80	Colon	3	1	0	-	-	-	-	3B	3	-	-	Adenocarcinoma	-	-	-
GSM76555	Colon	Female	Caucasian	Yes	Yes	Yes	80	Colon	3	0	0	-	-	-	-	2A	2	-	-	Adenocarcinoma	-	-	-
GSM76573	Colon	Male	Caucasian	Yes	No	Yes	80	Colon	3	0	0	-	-	-	-	2A	2	-	-	Adenocarcinoma	-	-	-
GSM76576	Colon	Female	Caucasian	No	No	No	60	Colon	2	0	0	-	-	-	-	1	2	-	-	Adenocarcinoma	-	-	-
GSM76583	Colon	Male	Caucasian	Yes	Yes	Yes	70	Colon	3	0	0	-	-	-	-	2A	2	-	-	Adenocarcinoma	-	-	-
GSM76598	Colon	Male	Caucasian	Yes	No	No	60	Colon	4	1	0	-	-	-	-	3B	1	-	-	Adenocarcinoma	-	-	-
GSM76607	Colon	Female	Caucasian	Yes	No	No	80	Colon	3	0	0	-	-	-	-	2A	3	-	-	Mucinous Carcinoma	-	-	-
GSM76611	Colon	Female	Caucasian	No	No	No	70	Colon	2	0	0	-	-	-	-	1	2	-	-	Adenocarcinoma	-	-	-
GSM76617	Colon	Female	Caucasian	No	Yes	No	60	Colon	3	0	0	-	-	-	-	2A	2	-	-	Adenocarcinoma	-	-	-
GSM76618	Colon	Male	African-America	Yes	No	No	80	Colon	3	0	0	-	-	-	-	2A	2	-	-	Adenocarcinoma	-	-	-
GSM76629	Colon	Male	African-America	No	Yes	Yes	60	Colon	1	0	0	-	-	-	-	1	2	-	-	Adenocarcinoma	-	-	-
GSM76639	Colon	Female	Caucasian	Yes	Yes	Yes	60	Colon	2	1	0	-	-	-	-	3A	3	-	-	Adenocarcinoma	-	-	-
GSM88945	Colon	Female	Caucasian	No	No	No	50	Colon	3	0	0	-	-	-	-	2A	2	-	-	Adenocarcinoma	-	-	-
GSM88963	Colon	Female	Caucasian	No	No	No	70	Colon	3	0	0	-	-	-	-	2A	2	-	-	Adenocarcinoma	-	-	-
GSM88968	Colon	Male	Caucasian	No	No	Yes	50	Colon	3	1	0	-	-	-	-	3B	2	-	-	Adenocarcinoma	-	-	-
GSM88999	Colon	Female	Caucasian	No	No	Yes	40	Colon	3	0	0	-	-	-	-	2A	2	-	-	Adenocarcinoma	-	-	-

GSM89002	Colon	Male	Caucasian	Yes	No	Yes	70	Colon	3	2	0	-	-	-	-	2A	3	-	-	Adenocarcinoma	-	-	-
GSM89004	Colon	Female	Caucasian	No	No	No	60	Colon	3	0	0	-	-	-	-	2A	2	-	-	Adenocarcinoma	-	-	-
GSM89007	Colon	Female	Caucasian	No	No	No	70	Colon	3	0	0	-	-	-	-	2A	2	-	-	Adenocarcinoma	-	-	-
GSM89026	Colon	Female	Caucasian	Yes	Yes	No	40	Colon	3	0	0	-	-	-	-	2A	2	-	-	Adenocarcinoma	-	-	-
GSM89037	Colon	Male	Caucasian	No	Yes	No	60	Colon	3	1	0	-	-	-	-	3B	2	-	-	Adenocarcinoma	-	-	-
GSM89044	Colon	Female	Caucasian	No	No	Yes	50	Colon	2	0	0	-	-	-	-	1	2	-	-	Adenocarcinoma	-	-	-
GSM89047	Colon	Male	Caucasian	Yes	No	Yes	50	Colon	3	0	0	-	-	-	-	2A	2	-	-	Adenocarcinoma	-	-	-
GSM89049	Colon	Male	Caucasian	No	No	Yes	80	Colon	3	0	0	-	-	-	-	2A	2	-	-	Adenocarcinoma	-	-	-
GSM89052	Colon	Male	Caucasian	No	No	Yes	30	Colon	3	0	0	-	-	-	-	2A	2	-	-	Mucinous Carcinoma	-	-	-
GSM89053	Colon	Male	Caucasian	Yes	Yes	No	60	Colon	4	1	0	-	-	-	-	3B	3	-	-	Mucinous Carcinoma	-	-	-
GSM89061	Colon	Female	Caucasian	No	Yes	Yes	60	Colon	3	0	0	-	-	-	-	2A	2	-	-	Adenocarcinoma	-	-	-
GSM89062	Colon	Male	Caucasian	Yes	No	No	60	Colon	3	0	0	-	-	-	-	2A	2	-	-	Adenocarcinoma	-	-	-
GSM89069	Colon	Female	Caucasian	No	No	Yes	60	Colon	3	1	0	-	-	-	-	3B	2	-	-	Adenocarcinoma	-	-	-
GSM89090	Colon	Female	Caucasian	No	No	Yes	60	Colon	3	1	0	-	-	-	-	3B	2	-	-	Adenocarcinoma	-	-	-
GSM89098	Colon	Male	Caucasian	Yes	Yes	Yes	40	Colon	3	2	0	-	-	-	-	3C	2	-	-	Adenocarcinoma	-	-	-

D. Non-liver metastatic cancer from primary colon cancer

AC	Src	Gender	Ethnic	TBC	Alc	FM	Age	PSite	P T	P N	P M	RL	R T	R N	R M	P Stage	P Grade	R Stage	R Grade	Histology	C T	C N	C M
GSM102484	Small Intestine	Female	Caucasian	No	No	Yes	70	Colon	-	-	-	Yes	X	X	1	-	-	-	-	Metastatic Adenocarcinoma	-	-	-
GSM137976	Bladder	Male	Caucasian	Yes	No	Yes	50	Colon	-	-	-	Yes	X	0	1	-	-	-	-	Metastatic Adenocarcinoma	-	-	-
GSM138047	Lung	Male	Caucasian	Yes	Yes	Yes	60	Colon	-	-	-	Yes	X	X	1	-	-	-	-	Metastatic Mucinous Carcinoma	-	-	-
GSM152592	Lung	Female	Caucasian	No	No	Yes	80	Colon	-	-	-	Yes	X	X	1	-	-	-	-	Metastatic Adenocarcinoma	-	-	-
GSM152709	Ovary	Female	Caucasian	No	Yes	No	50	Colon	3	2	1	-	-	-	-	4	2	-	-	Metastatic Mucinous Carcinoma	-	-	-
GSM152721	Ovary	Female	Caucasian	No	No	No	50	Colon	3	1	1	-	-	-	-	4	3	-	-	Mucinous Carcinoma	-	-	-
GSM231937	Ovary	Female	Caucasian	Yes	No	Yes	50	Colon	-	-	-	Yes	3	1	1	-	-	-	-	Metastatic Adenocarcinoma	-	-	-
GSM46828	Liver or Omentum	Female	American Indian	No	No	No	30	Colon	3	2	1	-	-	-	-	4	3	-	-	Metastatic Adenocarcinoma	-	-	-
GSM46946	Adrenal gland	Female	Caucasian	Yes	No	Yes	50	Colon	-	-	-	Yes	X	X	1	-	-	-	-	Metastatic Adenocarcinoma	-	-	-
GSM53056	Ovary	Female	Caucasian	No	No	Yes	40	Colon	-	-	-	-	-	-	-	-	-	-	-	Metastatic Adenocarcinoma	-	-	-
GSM76509	Ovary	Female	Caucasian	No	No	Yes	70	Colon	-	-	-	Yes	X	2	1	-	-	-	-	Metastatic Adenocarcinoma	-	-	-
GSM76540	Omentum	Female	Caucasian	No	No	Yes	60	Colon	4	1	1	-	-	-	-	4	3	-	-	Metastatic Mucinous Carcinoma	-	-	-
GSM76574	Lung	Female	Caucasian	Yes	No	Yes	50	Colon	-	-	-	Yes	X	X	1	-	-	-	-	Metastatic Adenocarcinoma	-	-	-
GSM89036	Ovary	Female	Caucasian	No	Yes	Yes	50	Colon	-	-	-	Yes	X	X	1	-	-	-	-	Metastatic Adenocarcinoma	-	-	-
