# Supplementary material for: Mining metastasis related genes by primary-secondary tumor comparisons from large-scale databases
Source: BMC Bioinformatics. 2009 Mar 19;10(Suppl 3):S2. doi: 10.1186/1471-2105-10-S3-S2 (PMC2665050; doi:10.1186/1471-2105-10-S3-S2)
Supplement: Additional file 2 — Six heat maps of inter-tumor class comparisons [file 1471-2105-10-S3-S2-S2.pdf]

Supplementary data 2. Six heat-maps of inter-tumor class comparisons.

Six heat maps from inter-class comparisons are shown here. Heat maps are drawn using the GSEA analysis tool (Subramanian, PNAS, 2005). In the heat maps, the genes with highly differently expressions are identified. Red color indicates up-regulation, while blue denotes down regulation. Definition of classes is same as in the main text.

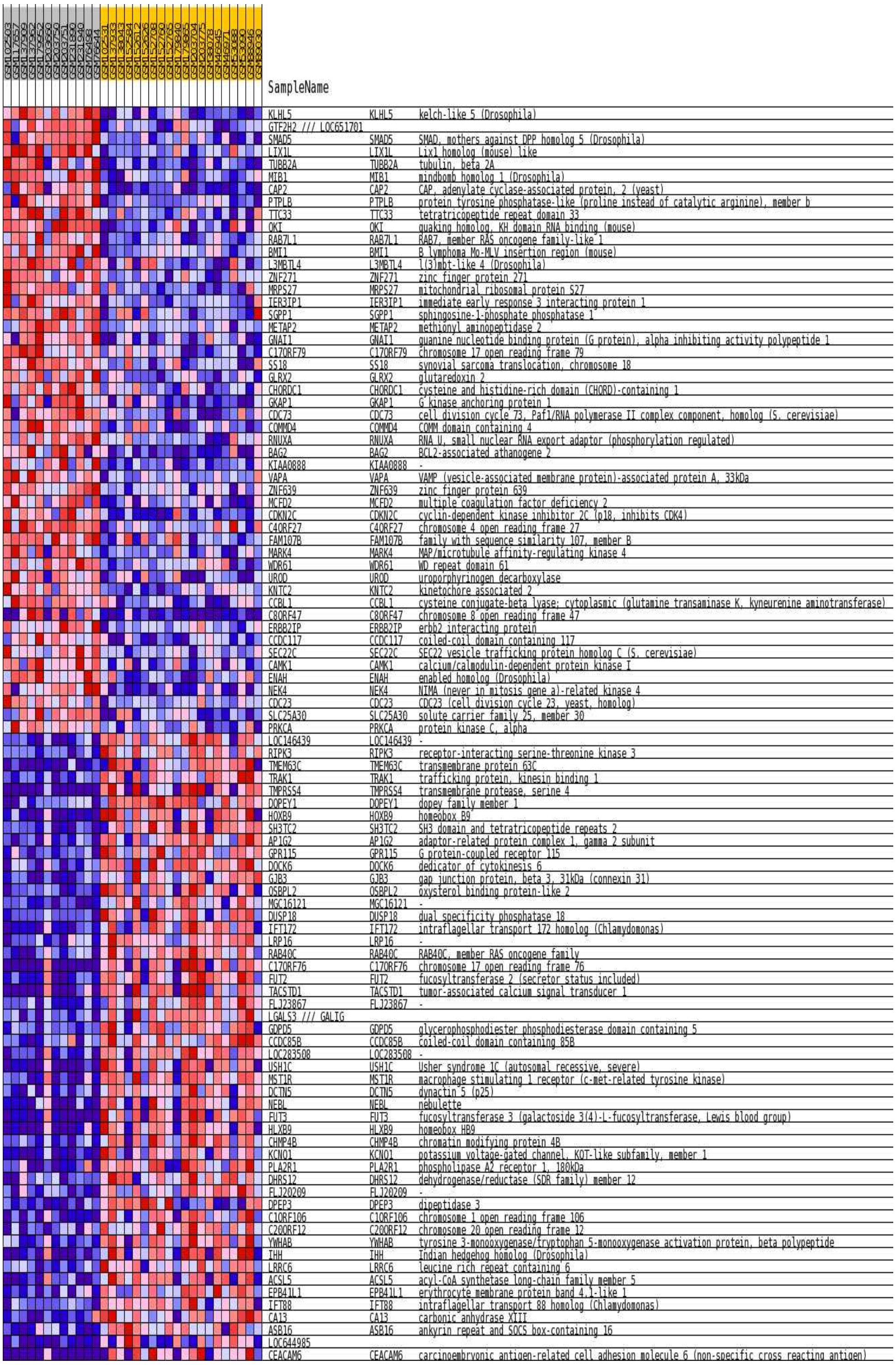

Heat map 1. Class A vs. B.

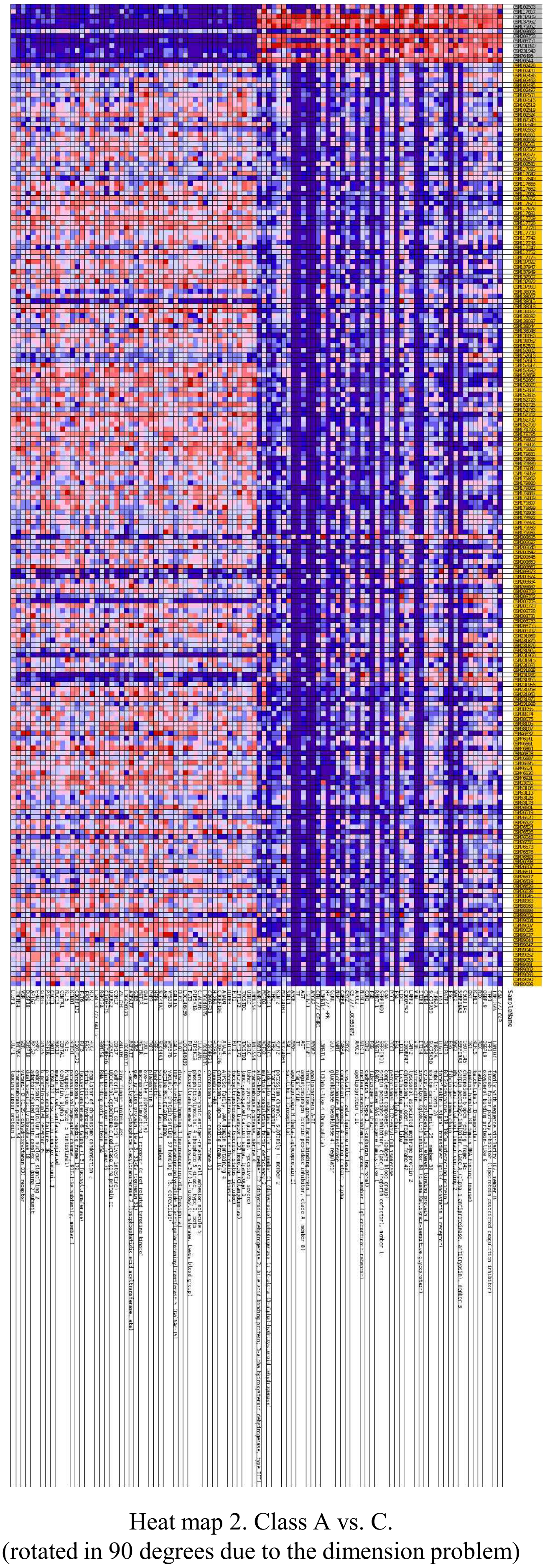

Heat map 2. Class A vs. C.
(rotated in 90 degrees due to the dimension problem)

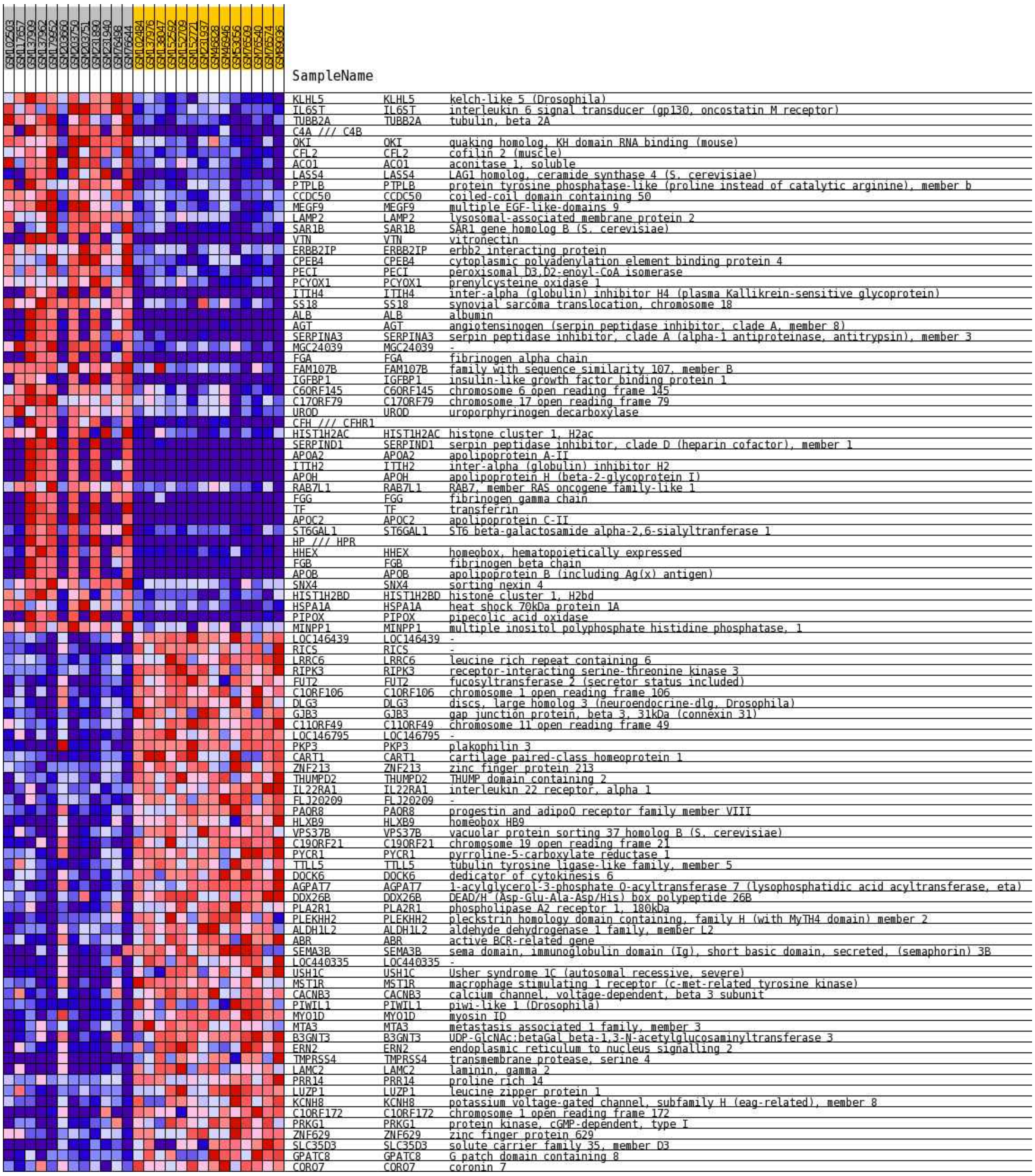

SampleName		
KLHL5	KLHL5	kelch-like 5 (Drosophila)
IL6ST	IL6ST	interleukin 6 signal transducer (gp130, oncostatin M receptor)
TUBB2A	TUBB2A	tubulin, beta 2A
C4A /// C4B		
QKI	QKI	quaking homolog, KH domain RNA binding (mouse)
CFL2	CFL2	cofilin 2 (muscle)
ACO1	ACO1	aconitase 1, soluble
LASS4	LASS4	LAG1 homolog, ceramide synthase 4 (S. cerevisiae)
PTPLB	PTPLB	protein tyrosine phosphatase-like (proline instead of catalytic arginine), member b
CCDC50	CCDC50	coiled-coil domain containing 50
MEGF9	MEGF9	multiple EGF-like-domains 9
LAMP2	LAMP2	lysosomal-associated membrane protein 2
SAR1B	SAR1B	SAR1 gene homolog B (S. cerevisiae)
VTN	VTN	vitronectin
ERBB2IP	ERBB2IP	erbb2 interacting protein
CPEB4	CPEB4	cytoplasmic polyadenylation element binding protein 4
PECI	PECI	peroxisomal D3,D2-enoyl-CoA isomerase
PCYOX1	PCYOX1	prenylcysteine oxidase 1
ITIH4	ITIH4	inter-alpha (globulin) inhibitor H4 (plasma Kallikrein-sensitive glycoprotein)
SS18	SS18	synovial sarcoma translocation, chromosome 18
ALB	ALB	albumin
AGT	AGT	angiotensinogen (serpin peptidase inhibitor, clade A, member 8)
SERPINA3	SERPINA3	serpin peptidase inhibitor, clade A (alpha-1 antiproteinase, antitrypsin), member 3
MGC24039	MGC24039	-
FGA	FGA	fibrinogen alpha chain
FAM107B	FAM107B	family with sequence similarity 107, member B
IGFBP1	IGFBP1	insulin-like growth factor binding protein 1
C6ORF145	C6ORF145	chromosome 6 open reading frame 145
C17ORF79	C17ORF79	chromosome 17 open reading frame 79
UROD	UROD	uroporphyrinogen decarboxylase
CFH /// CFHR1		
HIST1H2AC	HIST1H2AC	histone cluster 1, H2ac
SERPIND1	SERPIND1	serpin peptidase inhibitor, clade D (heparin cofactor), member 1
APOA2	APOA2	apolipoprotein A-II
ITIH2	ITIH2	inter-alpha (globulin) inhibitor H2
APOH	APOH	apolipoprotein H (beta-2-glycoprotein I)
RAB7L1	RAB7L1	RAB7, member RAS oncogene family-like 1
FGG	FGG	fibrinogen gamma chain
TF	TF	transferrin
APOC2	APOC2	apolipoprotein C-II
ST6GAL1	ST6GAL1	ST6 beta-galactosamide alpha-2,6-sialyltranferase 1
HP /// HPR		
HHEX	HHEX	homeobox, hematopoietically expressed
FGB	FGB	fibrinogen beta chain
APOB	APOB	apolipoprotein B (including Ag(x) antigen)
SNX4	SNX4	sorting nexin 4
HIST1H2BD	HIST1H2BD	histone cluster 1, H2bd
HSPA1A	HSPA1A	heat shock 70kDa protein 1A
PIPOX	PIPOX	pipecolic acid oxidase
MINPP1	MINPP1	multiple inositol polyphosphate histidine phosphatase, 1
LOC146439	LOC146439	-
RICS	RICS	-
LRRC6	LRRC6	leucine rich repeat containing 6
RIPK3	RIPK3	receptor-interacting serine-threonine kinase 3
FUT2	FUT2	fucosyltransferase 2 (secretor status included)
C1ORF106	C1ORF106	chromosome 1 open reading frame 106
DLG3	DLG3	discs, large homolog 3 (neuroendocrine-dlg, Drosophila)
GJB3	GJB3	gap junction protein, beta 3, 31kDa (connexin 31)
C11ORF49	C11ORF49	chromosome 11 open reading frame 49
LOC146795	LOC146795	-
PKP3	PKP3	plakophilin 3
CART1	CART1	cartilage paired-class homeoprotein 1
ZNF213	ZNF213	zinc finger protein 213
THUMPD2	THUMPD2	THUMP domain containing 2
IL22RA1	IL22RA1	interleukin 22 receptor, alpha 1
FLJ20209	FLJ20209	-
PAQR8	PAQR8	progestin and adipoQ receptor family member VIII
HLXB9	HLXB9	homeobox HB9
VPS37B	VPS37B	vacuolar protein sorting 37 homolog B (S. cerevisiae)
C19ORF21	C19ORF21	chromosome 19 open reading frame 21
PYCR1	PYCR1	pyrroline-5-carboxylate reductase 1
TTLL5	TTLL5	tubulin tyrosine ligase-like family, member 5
DOCK6	DOCK6	dedicator of cytokinesis 6
AGPAT7	AGPAT7	1-acylglycerol-3-phosphate O-acyltransferase 7 (lysophosphatidic acid acyltransferase, eta)
DDX26B	DDX26B	DEAD/H (Asp-Glu-Ala-Asp/His) box polypeptide 26B
PLA2R1	PLA2R1	phospholipase A2 receptor 1, 180kDa
PLEKHH2	PLEKHH2	pleckstrin homology domain containing, family H (with MyTH4 domain) member 2
ALDH1L2	ALDH1L2	aldehyde dehydrogenase 1 family, member L2
ABR	ABR	active BCR-related gene
SEMA3B	SEMA3B	sema domain, immunoglobulin domain (Ig), short basic domain, secreted, (semaphorin) 3B
LOC440335	LOC440335	-
USH1C	USH1C	Usher syndrome 1C (autosomal recessive, severe)
MST1R	MST1R	macrophage stimulating 1 receptor (c-met-related tyrosine kinase)
CACNB3	CACNB3	calcium channel, voltage-dependent, beta 3 subunit
PIWIL1	PIWIL1	piwi-like 1 (Drosophila)
MYO1D	MYO1D	myosin ID
MTA3	MTA3	metastasis associated 1 family, member 3
B3GNT3	B3GNT3	UDP-GlcNAc:betaGal beta-1,3-N-acetylglucosaminyltransferase 3
ERN2	ERN2	endoplasmic reticulum to nucleus signalling 2
TMPRSS4	TMPRSS4	transmembrane protease, serine 4
LAMC2	LAMC2	laminin, gamma 2
PRR14	PRR14	proline rich 14
LUZP1	LUZP1	leucine zipper protein 1
KCNH8	KCNH8	potassium voltage-gated channel, subfamily H (eag-related), member 8
C1ORF172	C1ORF172	chromosome 1 open reading frame 172
PRKG1	PRKG1	protein kinase, cGMP-dependent, type I
ZNF629	ZNF629	zinc finger protein 629
SLC35D3	SLC35D3	solute carrier family 35, member D3
GPATC8	GPATC8	G patch domain containing 8
CORO7	CORO7	coronin 7

Heat map 3. Class A vs. D.

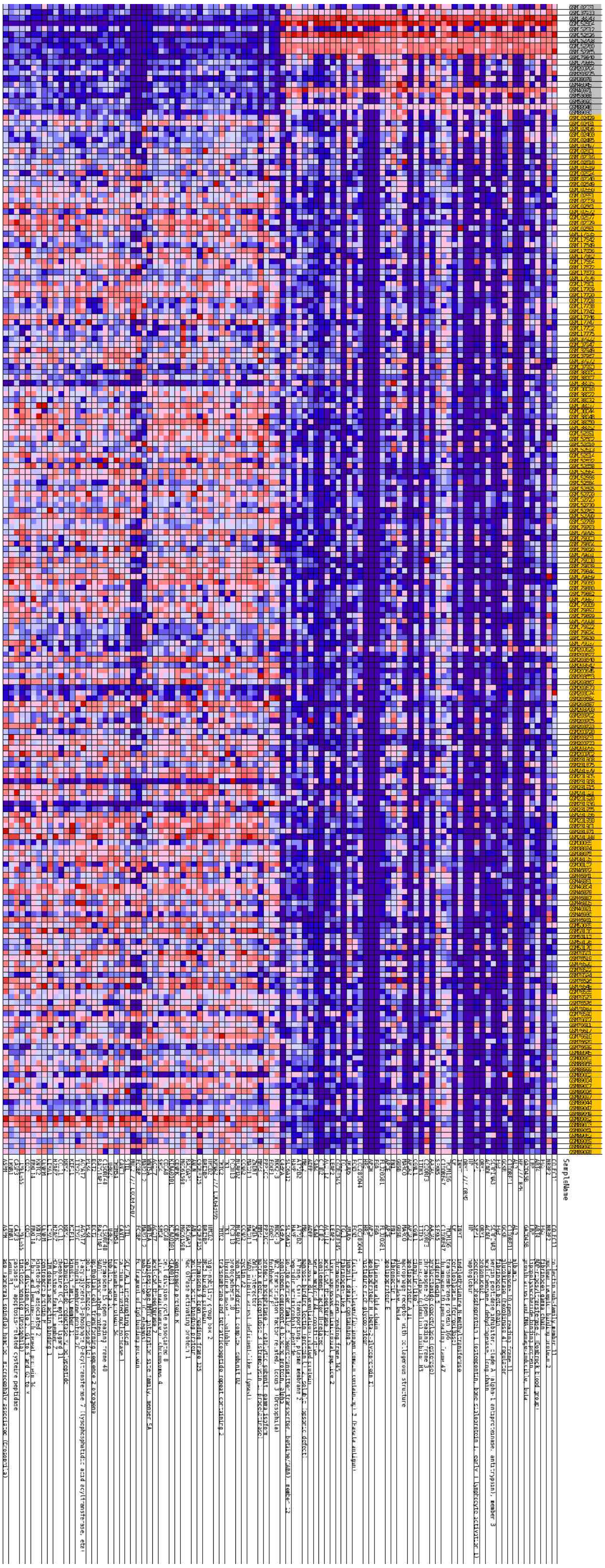

Heat map 4. Class B vs. C.

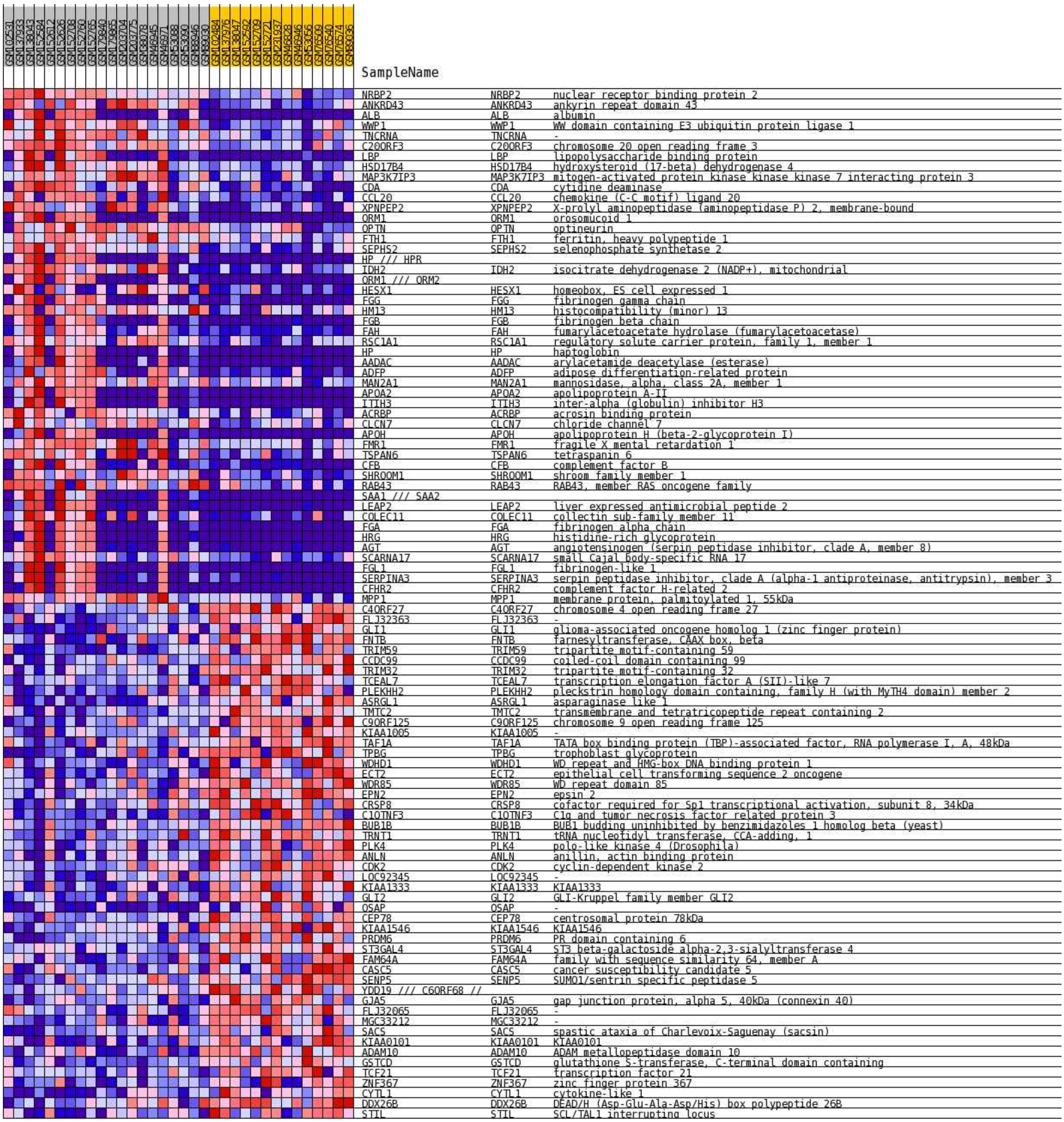

NRBP2	NRBP2	nuclear receptor binding protein 2
ANKRD43	ANKRD43	ankyrin repeat domain 43
ALB	ALB	albumin
WWP1	WWP1	WW domain containing E3 ubiquitin protein ligase 1
TNCRNA	TNCRNA	-
C20ORF3	C20ORF3	chromosome 20 open reading frame 3
LBP	LBP	lipopolysaccharide binding protein
HSD17B4	HSD17B4	hydroxysteroid (17-beta) dehydrogenase 4
MAP3K7IP3	MAP3K7IP3	mitogen-activated protein kinase kinase kinase 7 interacting protein 3
CDA	CDA	cytidine deaminase
CCL20	CCL20	chemokine (C-C motif) ligand 20
XPNPEP2	XPNPEP2	X-prolyl aminopeptidase (aminopeptidase P) 2, membrane-bound
ORM1	ORM1	orosomucoid 1
OPTN	OPTN	optineurin
FTH1	FTH1	ferritin, heavy polypeptide 1
SEPHS2	SEPHS2	selenophosphate synthetase 2
HP /// HPR		
IDH2	IDH2	isocitrate dehydrogenase 2 (NADP+), mitochondrial
ORM1 /// ORM2		
HESX1	HESX1	homeobox, ES cell expressed 1
FGG	FGG	fibrinogen gamma chain
HM13	HM13	histocompatibility (minor) 13
FGB	FGB	fibrinogen beta chain
FAH	FAH	fumarylacetoacetate hydrolase (fumarylacetoacetase)
RSC1A1	RSC1A1	regulatory solute carrier protein, family 1, member 1
HP	HP	haptoglobin
AADAC	AADAC	arylacetamide deacetylase (esterase)
ADFP	ADFP	adipose differentiation-related protein
MAN2A1	MAN2A1	mannosidase, alpha, class 2A, member 1
APOA2	APOA2	apolipoprotein A-II
ITIH3	ITIH3	inter-alpha (globulin) inhibitor H3
ACRBP	ACRBP	acrosin binding protein
CLCN7	CLCN7	chloride channel 7
APOH	APOH	apolipoprotein H (beta-2-glycoprotein I)
FMR1	FMR1	fragile X mental retardation 1
TSPAN6	TSPAN6	tetraspanin 6
CFB	CFB	complement factor B
SHROOM1	SHROOM1	shroom family member 1
RAB43	RAB43	RAB43, member RAS oncogene family
SAA1 /// SAA2		
LEAP2	LEAP2	liver expressed antimicrobial peptide 2
COLEC11	COLEC11	collectin sub-family member 11
FGA	FGA	fibrinogen alpha chain
HRG	HRG	histidine-rich glycoprotein
AGT	AGT	angiotensinogen (serpin peptidase inhibitor, clade A, member 8)
SCARNA17	SCARNA17	small Cajal body-specific RNA 17
FGL1	FGL1	fibrinogen-like 1
SERPINA3	SERPINA3	serpin peptidase inhibitor, clade A (alpha-1 antiproteinase, antitrypsin), member 3
CFHR2	CFHR2	complement factor H-related 2
MPP1	MPP1	membrane protein, palmitoylated 1, 55kDa
C4ORF27	C4ORF27	chromosome 4 open reading frame 27
FLJ32363	FLJ32363	-
GLI1	GLI1	glioma-associated oncogene homolog 1 (zinc finger protein)
FNTB	FNTB	farnesyltransferase, CAAX box, beta
TRIM59	TRIM59	tripartite motif-containing 59
CCDC99	CCDC99	coiled-coil domain containing 99
TRIM32	TRIM32	tripartite motif-containing 32
TCEAL7	TCEAL7	transcription elongation factor A (SII)-like 7
PLEKHH2	PLEKHH2	pleckstrin homology domain containing, family H (with MyTH4 domain) member 2
ASRGL1	ASRGL1	asparaginase like 1
TMTC2	TMTC2	transmembrane and tetratricopeptide repeat containing 2
C9ORF125	C9ORF125	chromosome 9 open reading frame 125
KIAA1005	KIAA1005	-
TAF1A	TAF1A	TATA box binding protein (TBP)-associated factor, RNA polymerase I, A, 48kDa
TPBG	TPBG	trophoblast glycoprotein
WDHD1	WDHD1	WD repeat and HMG-box DNA binding protein 1
ECT2	ECT2	epithelial cell transforming sequence 2 oncogene
WDR85	WDR85	WD repeat domain 85
EPN2	EPN2	epsin 2
CRSP8	CRSP8	cofactor required for Sp1 transcriptional activation, subunit 8, 34kDa
C1QTNF3	C1QTNF3	C1q and tumor necrosis factor related protein 3
BUB1B	BUB1B	BUB1 budding uninhibited by benzimidazoles 1 homolog beta (yeast)
TRNT1	TRNT1	tRNA nucleotidyl transferase, CCA-adding, 1
PLK4	PLK4	polo-like kinase 4 (Drosophila)
ANLN	ANLN	anillin, actin binding protein
CDK2	CDK2	cyclin-dependent kinase 2
LOC92345	LOC92345	-
KIAA1333	KIAA1333	KIAA1333
GLI2	GLI2	GLI-Kruppel family member GLI2
OSAP	OSAP	-
CEP78	CEP78	centrosomal protein 78kDa
KIAA1546	KIAA1546	KIAA1546
PRDM6	PRDM6	PR domain containing 6
ST3GAL4	ST3GAL4	ST3 beta-galactoside alpha-2,3-sialyltransferase 4
FAM64A	FAM64A	family with sequence similarity 64, member A
CASC5	CASC5	cancer susceptibility candidate 5
SENP5	SENP5	SUMO1/sentrin specific peptidase 5
YDD19 /// C6ORF68 //		
GJA5	GJA5	gap junction protein, alpha 5, 40kDa (connexin 40)
FLJ32065	FLJ32065	-
MGC33212	MGC33212	-
SACS	SACS	spastic ataxia of Charlevoix-Saguenay (sacsin)
KIAA0101	KIAA0101	KIAA0101
ADAM10	ADAM10	ADAM metallopeptidase domain 10
GSTCD	GSTCD	glutathione S-transferase, C-terminal domain containing
TCF21	TCF21	transcription factor 21
ZNF367	ZNF367	zinc finger protein 367
CYTL1	CYTL1	cytokine-like 1
DDX26B	DDX26B	DEAD/H (Asp-Glu-Ala-Asp/His) box polypeptide 26B
STIL	STIL	SCL/TAL1 interrupting locus

Heat map 5. Class B vs. D.

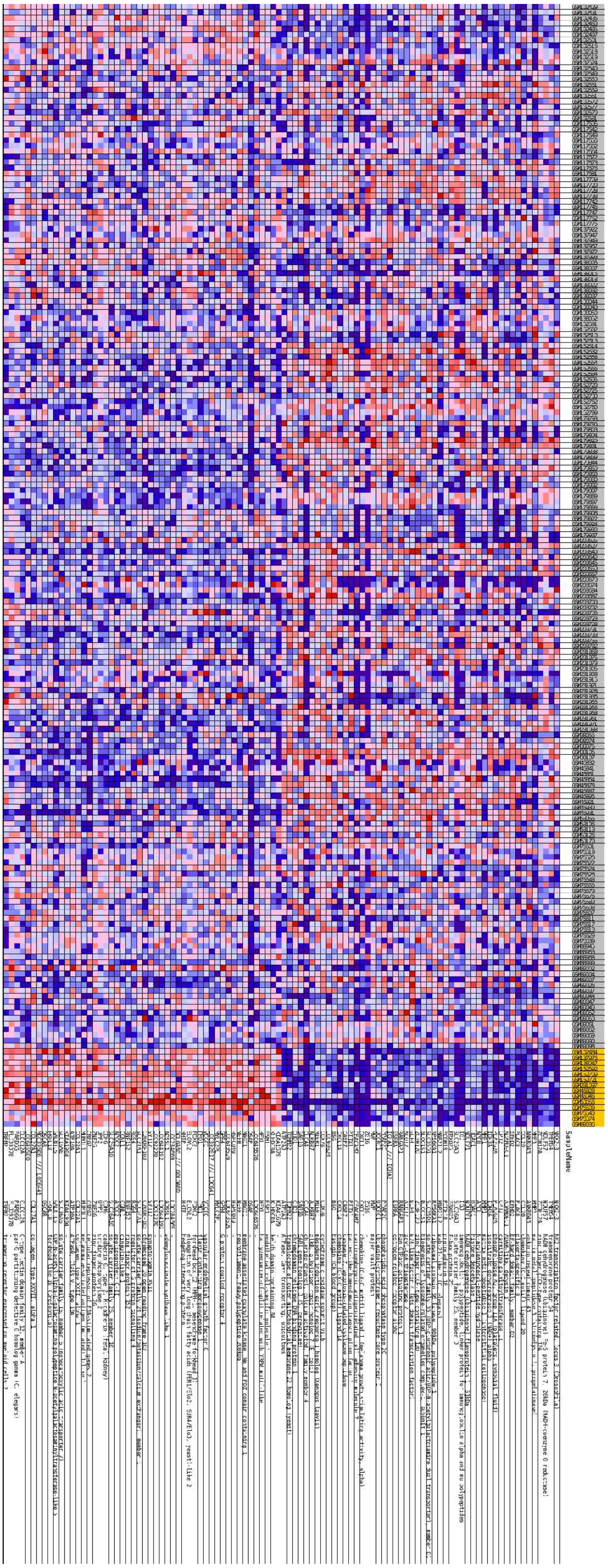

Heat map 6. Class C vs. D.
